# Widespread Higher Soil Respiration Rates at Nighttime Than Daytime Across Global Forest Ecosystems

**DOI:** 10.1111/gcb.70798

**Published:** 2026-03-13

**Authors:** Heng Huang, Jinyun Tang, Ben Bond‐Lamberty, Peter B. Reich, Thomas W. Crowther, Jinshi Jian, Kun Zhang, Lingli Liu, Jin Wu

**Affiliations:** ^1^ School of Ecology Shenzhen Campus of Sun Yat‐Sen University Shenzhen Guangdong China; ^2^ School of Biological Sciences and Institute for Climate and Carbon Neutrality The University of Hong Kong Hong Kong China; ^3^ Department of Climate Sciences, Earth and Environmental Sciences Area Lawrence Berkeley National Laboratory Berkeley California USA; ^4^ Joint Global Change Research Institute Pacific Northwest National Laboratory College Park Maryland USA; ^5^ Department of Forest Resources University of Minnesota St. Paul Minnesota USA; ^6^ Institute for Global Change Biology University of Michigan Ann Arbor Michigan USA; ^7^ Department of Environmental Systems Science ETH Zürich Zürich Switzerland; ^8^ State Key Laboratory of Soil and Water Conservation and Desertification Control Northwest A&F University Yangling China; ^9^ School of Geospatial Engineering and Science Sun Yat‐Sen University Zhuhai China; ^10^ State Key Laboratory of Vegetation and Environmental Change, Institute of Botany Chinese Academy of Sciences Beijing China; ^11^ China National Botanical Garden Beijing China; ^12^ University of Chinese Academy of Sciences Beijing China; ^13^ State Key Laboratory of Agrobiotechnology Chinese University of Hong Kong Hong Kong China

**Keywords:** carbon cycle, climate change, diel dynamics, Earth system model, forest, soil respiration, temperature sensitivity

## Abstract

Soil respiration (*R*_s_) is the second largest terrestrial carbon flux and therefore its temporal dynamics exert a significant influence on the soil carbon budget. While the seasonal and annual dynamics of *R*_s_ and its temperature sensitivity have been well documented, the diel *R*_s_ dynamics remain poorly understood. Earth system models (ESMs) typically assume a constant temperature response of *R*_s_ over the diel cycle, thereby predicting lower *R*_s_ at night than during the warmer daytime. Here, by analyzing extensive in situ *R*_s_ datasets from 36 global forest sites, we reveal an unexpectedly widespread pattern of higher nighttime than daytime *R*_s_, which is likely driven by the hourly temporal lag between recent photosynthetic assimilation and *R*_s_ associated with the transportation of recent photosynthates to the roots. Moreover, applying daytime *R*_s_‐temperature relationships systematically underestimates nighttime *R*_s_ by 2.5% to 28.7% across 31 sites, due to the significant diel difference in the temperature response of *R*_s_. However, ESMs predict lower *R*_s_ at night than during the day, largely resulting from the significant underestimation of nighttime root respiration dynamics. Our findings demonstrate significant diel *R*_s_ patterns across forest ecosystems, suggesting that daytime and nighttime *R*_s_ may respond distinctly to future climatic changes. Incorporating these diel dynamics is essential for improving predictions of terrestrial carbon‐climate feedbacks under global warming.

## Graphical abstract

Soil respiration is the second largest carbon flux between terrestrial ecosystems and the atmosphere, yet its diel dynamics remain poorly understood. By synthesizing continuous, in situ soil respiration measurements from forests worldwide, we reveal widespread higher soil respiration rates at night than during the day. The diel difference in the temperature response of soil respiration leads to up to a 28.7% underestimation of nighttime soil respiration across sites when using daytime‐based relationships. Our findings suggest that daytime and nighttime soil respiration in forests can experience distinct changes under climate warming, with significant impacts on the future trajectory of soil carbon emissions.

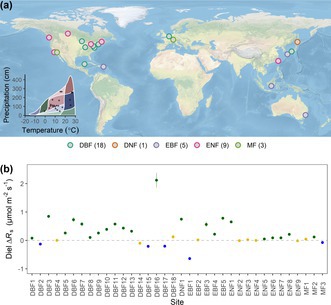

## 1 Introduction

Global soils contain nearly three times the amount of carbon (C) present in the atmosphere, making them the largest terrestrial C reservoir (Lehmann and Kleber 2015). Soil respiration (*R*_s_) constitutes the second largest terrestrial C flux, releasing 75–100 Pg C per year into the atmosphere via the activity of plant roots and soil microorganisms (Hashimoto et al. 2023; Bond‐Lamberty et al. 2024). Despite its crucial role in regulating the soil C budget and overall ecosystem C storage (Bond‐Lamberty et al. 2024), *R*_s_ cannot be observed directly through methods like eddy covariance towers or satellites, rendering it a highly uncertain component of the terrestrial C cycle (Warner et al. 2019). Improved understanding of how *R*_s_ responds to climate change is essential for accurately predicting future soil C dynamics and evaluating the resilience of the land C sink (Ruehr et al. 2023).

Extensive research has explored the seasonal and annual variability of *R*_s_ (Mo et al. 2005; Chen et al. 2014; Hursh et al. 2017) and its response to warming (Liang et al. 2024). Despite empirical evidence revealing significant differences in diel *R*_s_ patterns observed at the single‐site level across diverse ecosystems (e.g., Xia et al. 2009; Ruehr et al. 2010; Brændholt et al. 2017; Hu et al. 2016; Makita et al. 2018; del Gutiérrez Arroyo and Wood 2020; Chu et al. 2023; Fekadu et al. 2022; Liu et al. 2024), the general cross‐site patterns of diel (day‐night) differences in *R*_s_, especially in forests, remain largely unclear (Han et al. 2023). Previous studies have reported overall higher *R*_s_ during the day than at night (Liu et al. 2006; Ford et al. 2012), attributing this pattern primarily to fluctuations in soil temperature (*T*_s_) (Lloyd and Taylor 1994; Hirano 2005; Carey et al. 2016). In this study, we broadly evaluate both the questions at the heart of these reported findings and their interpretation, with a larger and more comprehensive dataset. For example, the idea that *R*_s_ is higher at day than night due to higher daytime *T*_s_ assumes that both the short‐term (minutes to hours) and long‐term (days to months) temperature sensitivity of *R*_s_ (*Q*_10_, the relative change in *R*_s_ for a 10°C increase in temperature) remains constant throughout the day and night. Such a constant *Q*_10_ assumption is often used in many current Earth system models (ESMs) to simulate *R*_s_ and its components, that is, root respiration (*R*_root_) and soil microbial respiration (*R*_h_) (Ito et al. 2020; Guenet et al. 2024). However, recent in situ observations in a temperate forest have challenged this view, revealing significant diel differences in *Q*_10_ (Han et al. 2023). These findings suggest that *R*_s_‐*T*_s_ relationships can vary considerably between day and night, and that overlooking these variations may introduce substantial uncertainties into global *R*_s_ flux estimates (Jian et al. 2018; Hashimoto et al. 2023; Bond‐Lamberty et al. 2024).

Investigating diel differences in *R*_s_ and its temperature response is thus both timely and significant, especially in light of the growing evidence of a faster increase in minimum nighttime temperatures compared to maximum daytime temperatures in many regions worldwide (Doan et al. 2022). This asymmetric warming can directly impact biological metabolism and affect larger‐scale C fluxes (Peng et al. 2013). Therefore, understanding the diel difference in *R*_s_ and its temperature response is of paramount importance, with direct relevance to reduce the high uncertainty in current ESMs' projection of soil C dynamics under climate change (Luo et al. 2016; Warner et al. 2019).

**FIGURE 1:**
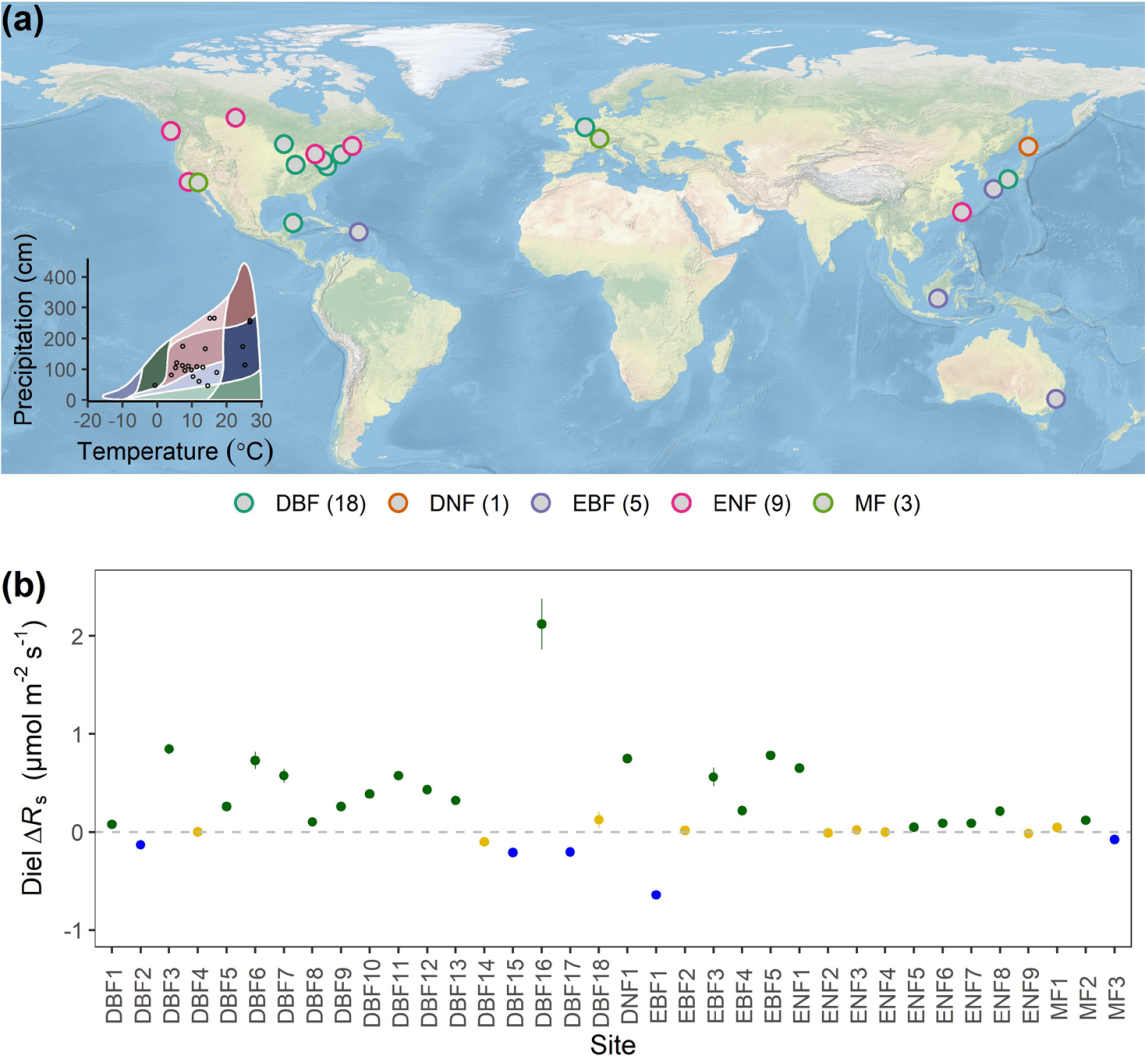
The diel patterns of soil respiration (*R*_s_) across global study sites. (a) Global map showing the geographic locations of forest sites. Two‐dimensional climate space superimposed onto Whittaker biomes. The number of sites for each forest type was shown. (b) The diel difference in soil respiration (∆*R*_s_ = nighttime *R*_s_—daytime *R*_s_, μmol m^−2^ s^−1^) across study sites. Positive values indicate higher nighttime values than daytime values. Dark‐green and blue points indicate significant positive and negative ∆*R*_s_ at each site (*p* < 0.05), while brown points indicate statistically nonsignificant diel difference (*p* ≥ 0.05). Error bars represent the standard error of the mean. Sites within each forest type were ordered sequentially based on an ascending gradient of site‐specific mean soil temperature. DBF, deciduous broadleaf forest; EBF, evergreen broadleaf forest; DNF, deciduous needleleaf forest; ENF, evergreen needleleaf forest; MF, mixed forest.

The growing availability of long‐term, continuous in situ *R*_s_ measurements provides an opportunity to systematically evaluate these diel differences and their temperature sensitivity across various sites. This study aims to explore the diel difference in *R*_s_ and *R*_s_‐*T*_s_ relationships, as well as the associated uncertainty in estimating nighttime *R*_s_ across global forest ecosystems. To achieve this, we utilize the global continuous soil respiration database (COSORE; Bond‐Lamberty et al. 2020), the global eddy covariance flux database (Pastorello et al. 2020), and ESM simulation outputs. Specifically, our research objectives are to (1) assess the consistency of diel patterns in *R*_s_ across global forest sites and the potential underlying mechanisms; (2) evaluate the performance of current ESMs in capturing diel variations in *R*_s_ and its components; and (3) quantify the uncertainty in nighttime *R*_s_ estimation when not accounting for diel difference in *R*_s_‐*T*_s_ relationships (e.g., using daytime *R*_s_‐*T*_s_ relationships for nighttime estimation). To tackle these questions, we focused on *R*_s_ data collected during the summer season (or equivalent in tropical forests), as it typically represents the peak season for *R*_s_ and offers a greater availability of measurements. Results of diel *R*_s_ dynamics for other seasons are provided in the Supporting Information, demonstrating similar findings especially in spring and autumn. We applied rigorous filtering procedures to focus on well‐characterized forest sites with sufficient daytime and nighttime soil respiration and temperature measurements, excluding data from manipulated experiments and potential outliers to ensure the robustness of data analysis. Following rigorous data preprocessing and quality control (detailed in Methods), our analysis included 36 sites across five forest types, as classified by the International Geosphere‐Biosphere Programme (IGBP; Figure 1a).

## 2 Materials and Methods

### 2.1 Investigating Diel *R*_s_ Differences Using the COSORE Database

In this study, we retrieved the sub‐hourly measurements of *R*_s_ (μmol m^−2^ s^−1^), *T*_s_ (°C), and soil water content (SWC, %) from the COSORE database to explore the diel patterns of *R*_s_ and its temperature response across forest ecosystems. The COSORE database comprises continuous and long‐term chamber‐based measurements of soil‐atmosphere greenhouse gas fluxes from 89 sites across various ecosystems (Bond‐Lamberty et al. 2020). Our analysis focused on the summer season, defined as June to August for the Northern Hemisphere and December to February for the Southern Hemisphere. Results for other seasons were provided in Figure S1. To ensure the robustness and reliability of our findings, we implemented a series of filtering criteria: (1) focus on forest sites: we concentrated on forest sites due to the limited availability of data from other land cover types; (2) comprehensive data requirements: only sites with records of both daytime and nighttime *T*_s_ and *R*_s_ were included in the study. Daytime and nighttime periods for each study site were determined by calculating the sunrise and sunset times for each day based on the Jet Propulsion Laboratory Development Ephemeris (Mahooti 2025); (3) exclusion of treatment data: data from warming or drought treatments were excluded to prevent confounding effects on *R*_s_; (4) measurement frequency thresholds: we aggregated the sub‐hourly measurements of *R*_s_, *T*_s_, and SWC to an hourly time scale to minimize the heterogeneity arising from variations in the measurement frequency both within and across sites. Months with fewer than 10 days of measurements were then excluded to ensure that the selected sites have sufficient and representative measurements. Similarly, days with fewer than 3 h of measurements in both daytime and nighttime periods were also excluded to ensure a robust comparison of *R*_s_ patterns between daytime and nighttime; (5) data quality control: non‐positive and outlier values of *R*_s_ for each dataset, defined as values outside the range [25% quantile—1.5 × interquartile range, 75% quantile +1.5 × interquartile range], were removed, though this did not affect the qualitative findings of this study.

Given the varied soil depths for *T*_s_ and SWC measurements across sites, we selected *T*_s_ and SWC data at a 5 cm depth, or the nearest available depth if 5 cm data were unavailable, to ensure consistency. The 5 cm depth was selected since this layer usually accounts for the majority of *R*_s_ relative to deeper soil profiles (Peng et al. 2009; Pries et al. 2017; Li et al. 2020). Our final dataset comprises 164,170 measurements of *R*_s_ from 36 forest sites, representing five IGBP (International Geosphere‐Biosphere Program) land cover types: deciduous broadleaf forest (DBF), evergreen broadleaf forest (EBF), deciduous needleleaf forest (DNF), evergreen needleleaf forest (ENF), and mixed forest (MF).

For each site, we calculated the daily mean daytime and nighttime *T*_s_, SWC, and *R*_s_ by averaging the corresponding hourly measurements during the daytime and nighttime periods and then used the Student's *t*‐test to examine the statistical significance of the diel differences in *T*_s_, SWC, and *R*_s_ across sites. In addition, we used the hourly measurement data to fit the daytime and nighttime *R*_s_‐*T*_s_ relationships using an exponential function, that is,

(1) $$R_{s}=R_{s0}e^{\beta T_{s}}$$

where *R*_s0_ and *β* are fitted constants. The temperature sensitivity of *R*_s_, denoted by *Q*_10_ ($Q_{10}=e^{10\beta }$), represents the relative change in *R*_s_ for a 10°C temperature increase. Because *R*_s_ is regulated by multiple processes operating at different time steps, this *Q*_10_ is intended as an integrated metric for long‐term average responses that include, but are not limited to temperature, given other drivers that co‐vary with temperature. To account for the effects of diel temperature variations on *R*_s_ patterns, we further tested the statistical significance of the difference in temperature‐adjusted *R*_s_ across sites by adjusting the raw daytime and nighttime *R*_s_ values to a common temperature using daytime and nighttime *Q*_10_ values, respectively, based on the following equation.

(2) $$R_{s}^{\mathrm{adj}}=R_{s}\times Q_{10}^{\left(\bar{T_{s}}-T_{s}\right)/10}$$

where $R_{s}^{\mathrm{adj}}$ is the temperature‐adjusted soil respiration (μmol m^−2^ s^−1^) and $\bar{T_{s}}$ is the site‐specific mean soil temperature (°C), which was calculated by averaging all soil temperature data during the summer period to fully reflect the long‐term climatic conditions of each site. Additionally, we conducted standardized major axis (SMA) regression analysis using the “lmodel2” function from the R package “lmodel2” (Legendre 2018) to compare daytime and nighttime *R*_s_ across study sites.

### 2.2 Evaluating ESMs' Ability to Simulate Diel Dynamics of *R*_s_

To evaluate the capacity of current ESMs in capturing diel patterns of *R*_s_, we ran two configurations of the ELM, the land surface component of the Earth system model E3SM (Burrows et al. 2020), to model global hourly dynamics of *R*_s_, *R*_root_, and *R*_h_ during the 2005–2014 period, which is well represented in the COSORE database and allows for a robust long‐term validation of ESM simulations against observational data. ELM simulates the complex land‐atmosphere interactions including energy, water, C, and nitrogen cycles. Like most current ESMs, ELM adopts a CENTURY‐like formulation of soil biogeochemistry (Koven et al. 2013). We took advantage of the two representations of nutrient competition dynamics in ELM to account for model structural uncertainty. Of the two ELM configurations, the Equilibrium Chemistry Approximation (ECA) configuration employs a mechanistic framework to model the complex competition network among plant roots, soil microbes, and abiotic protection mechanisms, explicitly representing interactions between multiple consumers and substrates. The ECA model uniquely features dynamic plant resource allocation, variable plant stoichiometry constrained by empirical observations (Kattge et al. 2020), and nutrient uptake driven by root functional traits instead of photosynthetic demand (Riley et al. 2018). The other configuration represents nutrient competition using the relative demand (RD) approach, where plants and soil biogeochemical processes compete equally for available nutrients. For both configurations, plant biomass growth is modeled as being driven by vegetation net primary productivity (NPP). However, the ECA configuration does not allow nighttime plant growth, whereas the RD configuration allows nighttime plant growth through some allowance of C deficit that is subsequently replenished by photosynthesis during daytime. We ran both model configurations using the land module only mode, driven by reanalysis climate data from the Global Soil Wetness Project phase 3 (Danger et al. 2008), and surface dataset as used in Zhu et al. (2020, 2024). The models were spinup using the recommended strategies of 200‐year accelerated spinup, 600‐year regular spinup, and transient simulation from 1850 to 2014. Hourly data of *R*_s_, autotrophic root respiration, heterotrophic respiration, and 10‐cm soil temperature were output for analysis.

From the hourly model outputs, we extracted the modelled values of *R*_s_, *R*_root_, and *R*_h_ from ECA and RD outputs for all 36 study sites. To ensure robustness, we calculated the site‐specific 10‐year average of daytime and nighttime means for *R*_s_, *R*_root_, and *R*_h_, respectively, during the 2005–2014 period. The daytime and nighttime periods for each pixel were determined by the time periods when GPP is equal to 0. We then applied SMA regression analysis to explore the diel difference in *R*_s_, *R*_root_, and *R*_h_, respectively.

### 2.3 Evaluating Biases in Predicting Nighttime *R*_s_ Using Daytime *R*_s_‐*T*_s_ Relationships

To quantify the potential biases in estimating nighttime *R*_s_ using daytime *R*_s_‐*T*_s_ relationships, we first estimated the model parameters *R*_s0_ and *β* from Equation (1) for the daytime periods at each site, and then used these daytime parameters along with nighttime *T*_s_ to yield nighttime *R*_s_ estimates. We then calculated the biases in nighttime estimation for each study site using the following equation:

(3) $$\text{Bias}\;\left(\%\right)=\frac{\left(R_{s}^{\mathrm{pre}}-R_{s}^{\mathrm{obs}}\right)}{R_{s}^{\mathrm{obs}}}\times 100\%$$

where $R_{s}^{\mathrm{pre}}$ and $R_{s}^{\mathrm{obs}}$ are the predicted and observed *R*_s_, respectively. The Student's *t*‐test was used to detect the statistical significance of the difference between $R_{s}^{\mathrm{pre}}$ and $R_{s}^{\mathrm{obs}}$ for each site. We roughly quantified, on a global scale, the approximate magnitude of error introduced by predicting nighttime *R*_s_ using daytime *R*_s_‐*T*_s_ relationships across different forest types in summer. This predictive error was determined by multiplying the difference between $R_{s}^{\mathrm{obs}}$ and $R_{s}^{\mathrm{pre}}$ by the average area of each forest type, derived from the MODIS land cover product MCD12C1. Additionally, we used a subset of COSORE sites (*n* = 8) with measurements of both *R*_s_ and *R*_h_ to examine the relative contributions of *R*_h_ and *R*_root_ to the biases in nighttime *R*_s_ estimation. The *R*_h_ time series data were measured primarily using the soil trenching approach (Bond‐Lamberty et al. 2020) and the *R*_root_ time series data were quantified as the difference between *R*_s_ and *R*_h_. We then calculated the biases in predicting nighttime *R*_root_ and *R*_h_ using daytime *R*_root_‐*T*_s_ relationships and *R*_h_‐*T*_s_ relationships, respectively.

### 2.4 Examining the Correlations Between *R*_s_ and GPP

We investigated the relationships of daytime and nighttime *R*_s_ with daily gross primary productivity (GPP) by leveraging the global (Pastorello et al. 2020) and AmeriFlux databases, which contains in situ eddy covariance measurements of CO_2_, water, and energy fluxes from different ecosystems across the globe. For this analysis, we selected forest sites (*n* = 11) with concurrent measurements of GPP from the FLUXNET and AmeriFlux networks and *R*_s_ from the COSORE database. Daily GPP estimates (g C m^−2^ day^−1^) based on the daytime partitioning method (Lasslop et al. 2010), which used both daytime and nighttime data for model parameterizations, were chosen for all study sites. We examined the relationships of daytime and nighttime *R*_s_ with daily GPP using the SMA regression analysis for each site to test whether nighttime *R*_s_ exhibited more elevated or more positive relationships with GPP than daytime *R*_s_ across sites. In addition, we analyzed the changes in the Pearson correlation coefficient between daily *R*_s_ and GPP as a function of a temporal lag between *R*_s_ and GPP, ranging from 0 to 45 days at each site. This time lag specifies the number of days prior to *R*_s_ measurements that the GPP data were used for the correlation analysis.

## 3 Results

### 3.1 Unexpected and Widespread Elevated Nighttime *R*_s_

**FIGURE 2:**
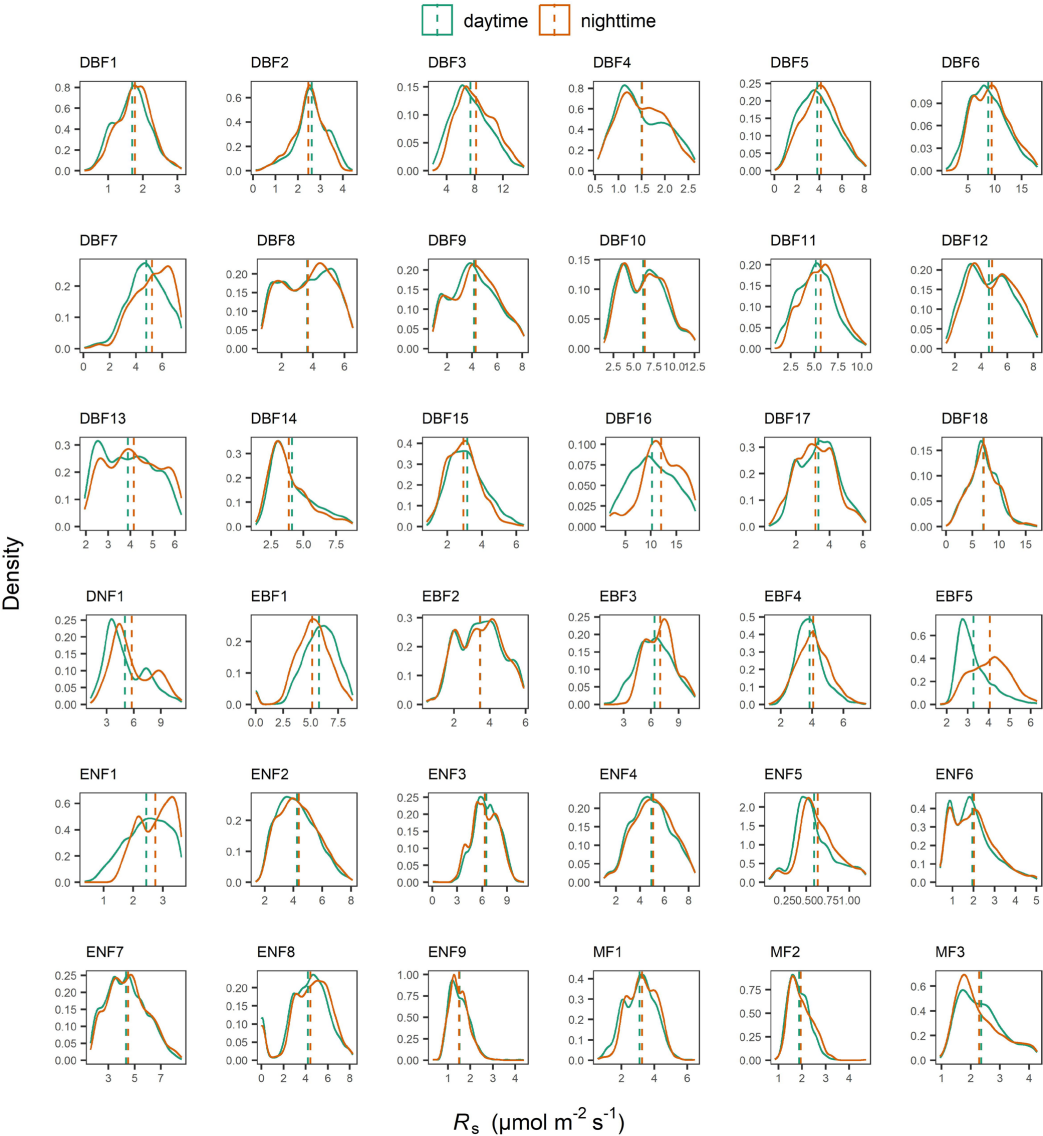
Density plots of daytime and nighttime *R*_s_ (μmol m^−2^ s^−1^) across forest sites. Green and orange solid lines represent daytime and nighttime density curves, respectively. The green and brown dashed lines represent the site‐specific mean values of daytime and nighttime *R*_s_, respectively.

**FIGURE 3:**
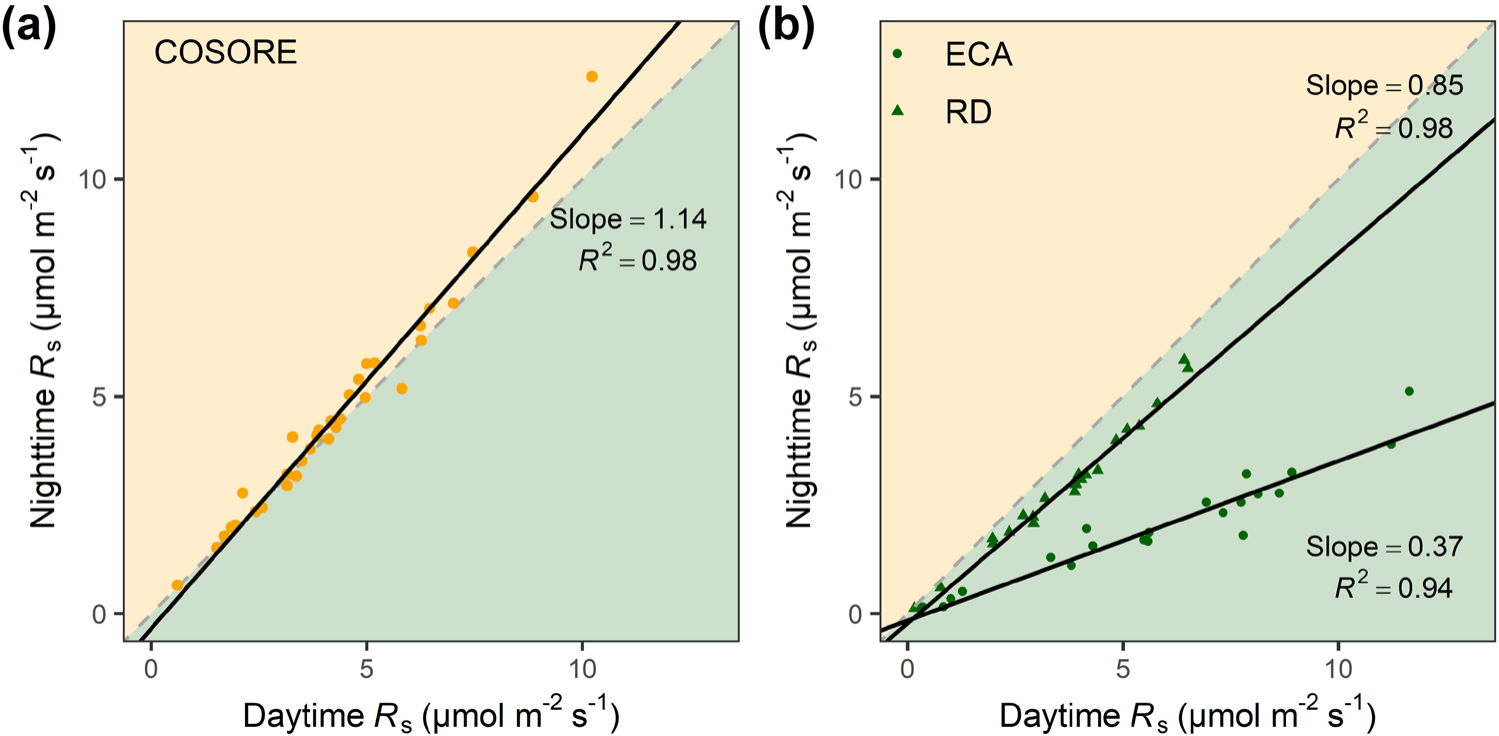
The comparison between daytime and nighttime *R*_s_ (μmol m^−2^ s^−1^). (a) The comparison between measured daytime and nighttime *R*_s_ across study sites. (b) The comparison between simulated daytime and nighttime *R*_s_ during 2005–2014 across study sites from the two models (i.e., ECA and RD). Each point represents the site‐specific mean value of *R*_s_. The dashed and solid lines represent the 1:1 relationships and linear regression results, respectively. Shaded orange areas represent values with higher nighttime than daytime and light green areas represent the opposite pattern.

We found an average diel difference of *R*_s_ (∆*R*_s_ = nighttime *R*_s_ − daytime *R*_s_) of 0.25 ± 0.08 μmol CO_2_ m^−2^ s^−1^ (mean ± SEM; roughly a 5.17% ± 1.40% difference) across all 36 forest sites examined in the COSORE database. Notably, a considerably larger proportion of these sites exhibited a significantly positive ∆*R*_s_ (*n* = 22, *p* < 0.05) than vice versa (*n* = 5, *p* < 0.05) (Figures 1b and 2), indicating a widespread (but not obligate) occurrence of higher nighttime *R*_s_ relative to daytime *R*_s_. All forest types on average exhibited a positive ∆*R*_s_, though the magnitude of this difference varied among forest types. Mixed forests (MF) had the smallest difference (0.03 ± 0.06 μmol CO_2_ m^−2^ s^−1^), whereas deciduous broadleaf forests (DBF) had the largest (0.34 ± 0.13 μmol CO_2_ m^−2^ s^−1^), followed by evergreen broadleaf forests (EBF: 0.19 ± 0.25 μmol CO_2_ m^−2^ s^−1^) and evergreen needleleaf forests (ENF: 0.12 ± 0.07 μmol CO_2_ m^−2^ s^−1^). In addition, ∆*R*_s_ was overall larger for sites with higher *R*_s_ as evidenced by a bivariate regression analysis (slope = 1.14, 95% CI = 1.08 to 1.21, *R* ^2^ = 0.98, Figure 3a).

We then examined the potential drivers of the observed diel *R*_s_ dynamics. A significant variation in ∆*T*_s_ (nighttime *T*_s_ − daytime *T*_s_) was observed across the 36 sites, ranging from −2.34 ± 0.07 to 2.52°C ± 0.07°C (cross‐site average: 0.07°C), with 19 and 13 sites exhibiting a significantly positive and negative ∆*T*_s_, respectively (all *p* < 0.05, Figure S2a). In contrast, there was little or no significant diel difference in soil water content (SWC, %) (Figure S2b), leading to similar daytime and nighttime *R*_s_‐SWC relationships (Figure S3). To assess whether the observed diel *R*_s_ difference was solely due to differences in *T*_s_ between day and night, we adjusted both daytime and nighttime *R*_s_ to the site‐specific mean soil temperature (*R*_s_^adj^) based on the site‐specific daytime and nighttime *Q*_10_ value, respectively (see Equation (1) in Methods). We found consistently higher nighttime *R*_s_^adj^ compared to daytime *R*_s_^adj^ across sites (Figure S4), suggesting that diel *R*_s_ dynamics were not primarily driven by the commonly considered ∆*T*_s_. In addition, both daytime and nighttime *Q*_10_ values were not significantly correlated with *T*_s_ and SWC (all *p* > 0.05, Figure S5).

### 3.2 Current ESM Underestimation of Nighttime *R*_s_

To assess the accuracy of ESMs in capturing elevated nighttime respiration observed in our analysis, we simulated global hourly *R*_s_, *R*_root_, and *R*_h_ for 2005–2014 using the land component of the Earth system model E3SM. We employed two model configurations, representing contrasting approaches to simulate nutrient dynamics: the equilibrium chemistry approximation (ECA) and the relative demand (RD) formulation (see Methods). Both configurations, representative of how *R*_s_ is typically represented in existing Earth system models (ESMs), were simulated at a half‐hourly time step to capture the full diel cycle of C and water fluxes. These long‐term simulations enabled a comprehensive evaluation of ESM outputs against empirical *R*_s_ data. Contrary to empirical observations, both ECA and RD simulations produced significantly lower nighttime *R*_s_ compared to daytime *R*_s_ (slope = 0.37 and 0.85, 95% CI = 0.34 to 0.40 and 0.81 to 0.90, *R* ^2^ = 0.94 and 0.98, both *p* < 0.01, respectively, Figure 3b). Across all sites, ECA‐ and RD‐estimated nighttime *R*_s_ were on average 65.6% ± 1.2% and 21.2% ± 0.8% lower than daytime *R*_s_, respectively. This discrepancy primarily stemmed from the significantly underestimated nighttime *R*_root_ simulated by both ECA (slope = 0.001, *p* > 0.05) and RD (slope = 0.71, 95% CI = 0.64 to 0.78, *R* ^2^ = 0.92, *p* < 0.01, Figure S6a), whereas *R*_h_ exhibited a less pronounced day‐night difference across both models (Figure S6b).

### 3.3 Substantial Bias in Nighttime *R*_s_ Prediction Using Daytime *R*_s_‐*T*_s_ Relationships

**FIGURE 4:**
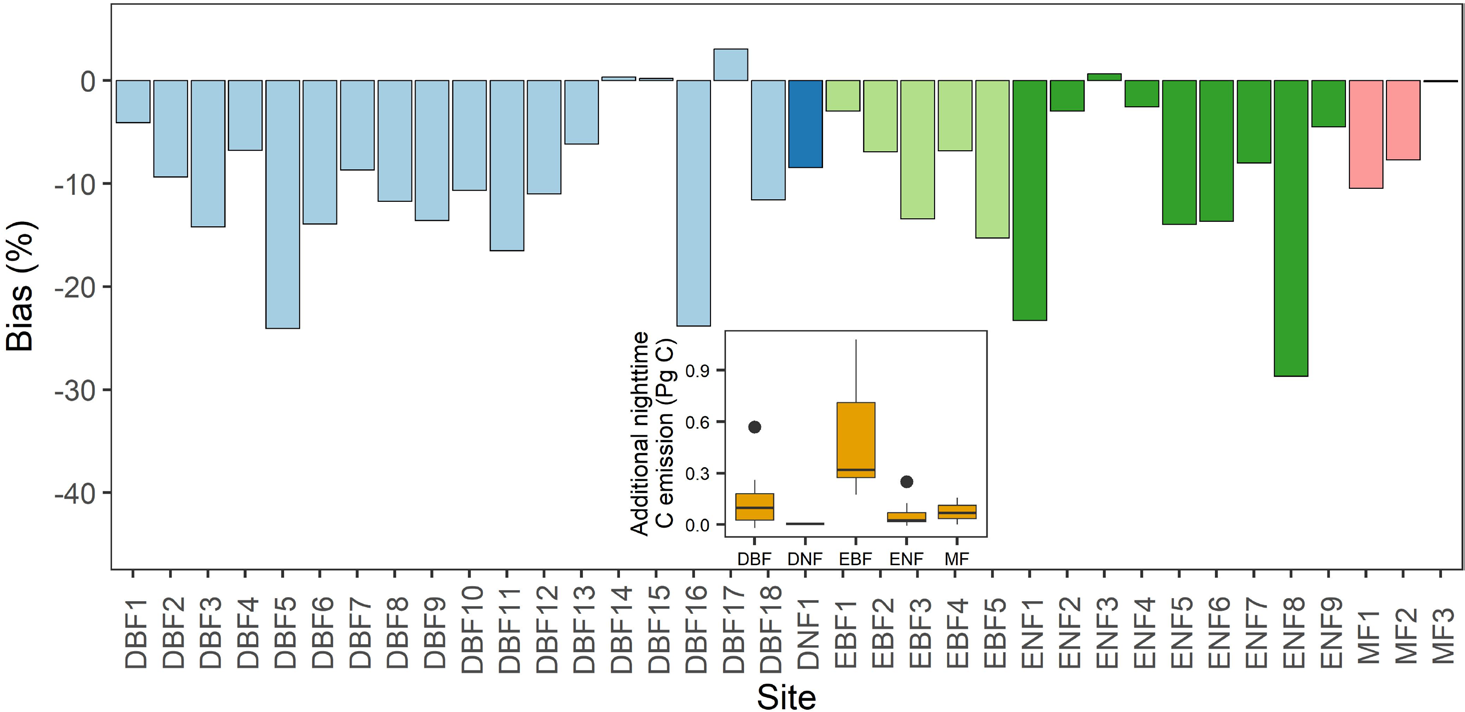
The bias in estimating nighttime *R*_s_ (%) using daytime *R*_s_‐*T*_s_ relationships. The bias percentage was calculated as $\left(R_{s}^{\mathrm{pre}}-R_{s}^{\mathrm{obs}}\right)/R_{s}^{\mathrm{obs}}\times 100\%$, where $R_{s}^{\mathrm{pre}}$ is the predicted nighttime *R*_s_ using daytime *R*_s_‐*T*_s_ relationships and $R_{s}^{\mathrm{obs}}$ is the observed nighttime *R*_s_. The bias is represented by colored bars corresponding to forest type as follows: Light blue (DBF), dark blue (DNF), light green (EBF), dark green (ENF), and pink (MF). The inset graph shows the additional nighttime C emission during the summer with respect to the predicted nighttime *R*_s_ based on daytime *R*_s_‐*T*_s_ relationships.

**FIGURE 5:**
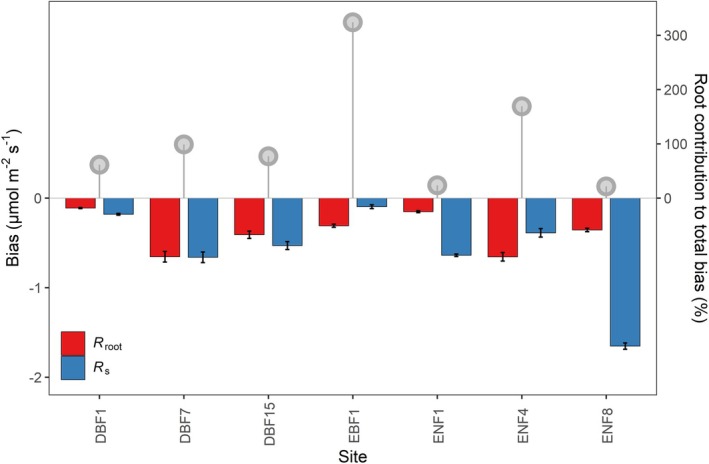
The bias in estimating nighttime *R*_s_ and root respiration (*R*_root_) based on their relationships with temperature during the day, respectively. The red and blue bars represent biases in *R*_root_ and *R*_s_ estimation, respectively. The grey points represent the percentage contribution of roots to *R*_s_ bias for different sites.

Given the notable differences in the parameters of *R*_s_‐*T*_s_ relationships between day and night (Figure S7, Table S1), we investigated the extent of bias when using daytime *R*_s_‐*T*_s_ relationships to estimate nighttime *R*_s_ across study sites. Among 36 sites, we found that 31 sites exhibited significant underestimation of nighttime *R*_s_ when daytime relationships were applied, with the underestimation ranging from 2.5% to 28.7% (Figures 4 and S8). These underestimations were mainly due to greater underestimation of nighttime *R*_root_ than *R*_h_, a pattern consistently observed across most sites (Figure 5). Notably, in the three DBF sites, bias in *R*_root_ prediction accounted for 61.4% to 98.9% of the bias in *R*_s_ prediction (Figure 5). When extrapolated to the global scale using the average global area of each forest type, applying the daytime *R*_s_‐*T*_s_ relationships to predict nighttime *R*_s_ could result in an average summertime underestimation of nighttime *R*_s_ by 0.51, 0.13, 0.06, and 0.07 Pg C on average for EBF, DBF, ENF, and MF, respectively.

## 4 Discussion

These results collectively provide the first global‐scale evidence for the widespread higher nighttime than daytime *R*_s_ in forest ecosystems during the summer season (Figure 1b). Notably, our synthesized cross‐site analysis aligns with recent work showing a higher nighttime *R*_s_ in a temperate forest (Han et al. 2023) but contrasts with most prior single‐site studies that reported higher daytime *R*_s_ due to warmer temperatures during the day across different ecosystems (Liu et al. 2006; Ford et al. 2012; Hu et al. 2016; Chu et al. 2023; Fekadu et al. 2022). This indicates the potentially important role of previously overlooked factors other than temperature in regulating diel *R*_s_ dynamics (Makita et al. 2018). Here, we propose two potential mechanisms underlying the observed widespread higher *R*_s_ at night than during the day. The first is related to the hourly‐scale lag between the assimilation and movement of new photosynthetic C and soil respiration (Baldocchi et al. 2006; Kuzyakov and Gavrichkova 2010; Han et al. 2014). In fact, recent photosynthates usually serve as a primary C source for *R*_s_ in forest ecosystems (e.g., Tang et al. 2005; Vargas and Allen 2008; Heinemeyer et al. 2012). Previous studies have reported that the time lag between photosynthesis and *R*_s_ is approximately 6–8 h in a mixed forest (Ruehr et al. 2010) and 7–12 h in an oak‐grass savanna (Tang et al. 2005). Consequently, greater rates of C fixation via daytime photosynthesis generally leads to an increased supply of recent photosynthates available to the roots. Due to this hourly temporal lag, the subsequent enhancement in the metabolic activities of roots and also soil microorganisms (i.e., rhizosphere priming effect; Kuzyakov and Cheng 2001) was observed to be significantly greater during the subsequent nighttime period than during the daytime (Vargas and Allen 2008). In addition, the time required for microbial decomposition of soil organic carbon can further contribute to the lagged CO_2_ efflux (Mencuccini and Hölttä 2010; Liu et al. 2024). This mechanism is at least partially supported by the evidence that (1) nighttime *R*_s_ showed more elevated and/or more positive relationships with GPP than daytime *R*_s_ across various forest sites (Figure S9); and (2) the correlation coefficient between daily *R*_s_ and GPP changed across different time lags, with the peak coefficient value varying across sites (Figure S10). These results are consistent with previous work showing positive dependence of *R*_s_ on photosynthesis (Xia et al. 2009). Furthermore, the magnitude of the diel difference in *R*_s_ in spring was similar to that in summer, both of which were greater than those observed in autumn and winter (Figure S1). This trend is likely attributed to the relatively higher photosynthetic activity in spring and summer, which consequently leads to increased allocation of recent photosynthates to belowground processes (Han et al. 2023). The second hypothesis concerns root xylem CO_2_ flux, as evidenced by empirical observations suggesting that a significant portion of CO_2_ respired by tree roots during the day is transported to the shoot via the transpiration stream (Aubrey and Teskey 2009; Bloemen et al. 2013, 2016). This upward transport can result in a lower observed daytime *R*_s_ and therefore contribute to the appearance of higher *R*_s_ at night.

Nevertheless, we acknowledge that the inherent disturbances associated with the long‐term deployment of automated chamber systems, such as weeding and microclimatic changes, may introduce systematic biases influencing diel *R*_s_ dynamics. In addition, empirical evidence suggests a potential overestimation of soil *R*_s_, especially at night, due to low atmospheric turbulence (Schneider et al. 2009; Brændholt et al. 2017), which is largely associated with the disruption of stratified air layers above the soil surface caused by the chamber movement during measurements (Görres et al. 2016). In fact, some datasets in the COSORE database have at least partially addressed this issue by utilizing fans or specialized designs to enhance air mixing or by discarding initial measurements to avoid closure effects. Crucially, the observed pattern of generally higher nighttime persists despite the inclusion of these mitigating approaches, underscoring the robustness of our results. Nevertheless, the overall impact of atmospheric turbulence on *R*_s_ estimation remains inadequately understood and addressed in current studies. Our study highlights the need of future experimental efforts that minimize these methodological artifacts and also integrate direct measurements of photosynthate allocation and root CO_2_ transport processes alongside concurrent monitoring of plant community structure and functions to comprehensively investigate the relative contributions of these physiological processes to diel *R*_s_ dynamics.

We observed higher nighttime than daytime *T*_s_ across a large subset of our study sites (Figure S2a), which may be attributed to (1) soil thermal inertia that allows soil to retain heat absorbed during the day (Cheruy et al. 2017) and (2) reduced radiative cooling, whereby forest canopies absorb a portion of nocturnal longwave radiation emitted by the soil surface and reflect it back towards the ground (D'Odorico et al. 2013). However, accounting for diel temperature variation did not alter diel *R*_s_ patterns across sites (Figure S4). According to Equation (1), diel *R*_s_ dynamics can also arise from the combined influence of diel differences in the reference soil respiration (*R*_s0_) and temperature sensitivity (*Q*_10_). For instance, higher nighttime *R*_s_ can be attributed to a higher *R*_s0_ and/or a greater *Q*_10_ at night than during the day. Indeed, we observed significant diel differences in *R*_s_‐*T*_s_ relationships across sites, as reflected by the diel variations in the numerical values of *Q*_10_ and *R*_s0_ (Table S1). While the negative cross‐site relationship between long‐term *Q*_10_ and temperature has been well recognized (Xu et al. 2015; Li et al. 2020), our cross‐site analysis revealed that both long‐term daytime and nighttime *Q*_10_ values were not significantly correlated with *T*_s_ or SWC (all *p* > 0.05, Figure S5). This implies that long‐term *Q*_10_ is not solely driven by climatic factors, but may instead reflect a complex interaction among biotic and abiotic drivers such as vegetation productivity and substrate quality (Davidson and Janssens 2006; Haaf et al. 2021). In addition, despite similar *R*_s_‐SWC relationships between day and night, SWC appears to exhibit a more pronounced influence on diel *R*_s_ dynamics under extreme wet and dry conditions (Figure S3), which might be associated with the effect of extreme SWC conditions on vegetation production and photosynthate allocation (Phillips et al. 2011).

By representing the allocation of new photosynthates to plant organs as a function of NPP (Lawrence et al. 2019), the ECA configuration does not allow root growth at night. As a result, nighttime root respiration only reflects maintenance respiration, and therefore nighttime *R*_s_ is dominated by soil microbial processes. Meanwhile, the RD configuration allows nighttime root growth based on a negative C storage pool that is only replenished during daytime, and this repletion acts to lower daytime growth respiration. Since empirical work indicates that plants continue root growth and nutrient uptake at night (Schimel et al. 1989; Riley et al. 2018; Tang and Riley 2021), these two representative model configurations and other similar models likely yield incorrect estimates of nighttime *R*_root_ as well as the diel cycle of *R*_root_ and its temperature sensitivity, as evidenced by the inconsistency in the diel *R*_root_ patterns between model predictions (Figure S6a) and empirical observations (Figure S11). The uncertainty surrounding *R*_root_ is further compounded by the challenge of partitioning *R*_s_ measurements into *R*_root_ and *R*_h_ components (Tang et al. 2019), as well as varying estimates of the contributions of *R*_root_ to *R*_s_, ranging from 10% to 90% depending on ecosystem types and climate conditions (Hanson et al. 2000). These factors result in yet poorly understood global spatial and temporal patterns of *R*_root_ (Ballantyne et al. 2017) and introduce large uncertainty in ESM simulations of the soil C budget (Tang et al. 2019).

In contrast to the observed diel difference in soil *Q*_10_ across sites (Table S1), many ESMs assume the same *Q*_10_ for both above‐ and below‐ground plant respiration and do not account for diel *Q*_10_ differences (Conant et al. 2011; Oleson et al. 2013; García‐Palacios et al. 2021), which may significantly increase uncertainty in simulating diel *R*_s_ dynamics. In addition, we also discovered a significant diel difference in the reference soil respiration (*R*_s0_) (see Equation (1); Figure S7; Table S1), which represents *R*_s_ at a reference temperature and therefore captures the responses of *R*_s_ to key factors other than temperature such as substrate availability and microbial biomass (Buchmann 2000). This finding contradicts the common assumption of a constant *R*_s0_ between day and night (Oleson et al. 2013) and highlights the potential uncertainty introduced by this assumption in current ESMs. This diel difference can also contribute to the observed elevated nighttime *R*_s_ and may be partially explained by the increased substrate availability at night due to the transport of recent photosynthetic C from leaves to roots (Baldocchi et al. 2006; Kuzyakov and Gavrichkova 2010; Han et al. 2014). However, more in‐depth analysis and mechanistic exploration is needed by future studies.

Our findings suggest a significant systematic underestimation of nighttime *R*_s_ when extrapolated using daytime *R*_s_‐*T*_s_ relationships (Figure 4). In fact, empirical studies usually extrapolate daytime *R*_s_ observations (e.g., from the morning period) to infer daily‐scale *R*_s_ when continuous *R*_s_ measurements from automated chamber systems are unavailable (Cueva et al. 2017). However, this approach can introduce significant uncertainty when *R*_s_ and its temperature response exhibit different diel patterns (Savage and Davidson 2003; Han et al. 2023). The availability of temporally‐continuous in situ *R*_s_ measurements across diverse ecosystems remains limited (Bond‐Lamberty et al. 2020), especially compared to other ecosystem‐scale C flux databases such as FLUXNET (Pastorello et al. 2020). The observed diel differences in *R*_s_ thus highlight the critical need for expanding *R*_s_ measurement networks to better understand the day‐night difference in *R*_s_ and its abiotic/biotic drivers across diverse ecosystems. Such expansion is vital not only for accurately quantifying the relative contribution of *R*_s_ to the overall soil C budget across various timescales, but also supporting evidence‐based soil C management and climate mitigation strategies.

Our study reveals widespread elevated nighttime *R*_s_ across global forest ecosystems, challenging the conventional view that *R*_s_ responds uniformly to temperature changes throughout day and night. Although previous studies have emphasized temperature and SWC as key drivers of *R*_s_ (Rytter 2013; Han et al. 2023; Liang et al. 2024), suggesting their potential to explain day‐night differences in *R*_s_, our analysis highlights the important role of diel difference in the temperature response of *R*_s_ in influencing the diel dynamics of *R*_s_ in forests. Together with evidence of asymmetric climate warming between day and night (Doan et al. 2022; Liu et al. 2024), our findings suggest that daytime and nighttime *R*_s_ in forests can experience distinct changes, impacting the future trajectory of soil C emissions and many other ecological processes related to the soil C turnover (Bond‐Lamberty et al. 2024). We acknowledge that other ecosystems, such as grasslands, typically exhibit greater diel soil temperature fluctuations and faster C turnover than forests, which may result in distinct diel *R*_s_ patterns that warrant further investigation. Enhancing the mechanistic understanding and model representation of diel dynamics of *R*_s_ across ecosystems and the potentially distinct diel patterns of its components (i.e., *R*_root_ and *R*_h_) is crucial for more accurate predictions of the temporal dynamics of soil C emissions under climate change.

## Author Contributions

H.H.: conceptualization, investigation, methodology, validation, visualization, writing – original draft, writing – review and editing. J.T.: investigation, methodology, validation, visualization, writing – review and editing. B.B.‐L.: conceptualization, methodology, validation, visualization, writing – review and editing. P.B.R.: conceptualization, methodology, validation, visualization, writing – review and editing. T.W.C.: methodology, validation, visualization, writing – review and editing. J.J.: methodology, investigation, writing – review and editing. K.Z.: formal analysis, investigation, writing – review and editing. L.L.: methodology, supervision, writing – review and editing. J.W.: conceptualization, project administration, resources, supervision, writing – review and editing.

## Funding

This work was supported by the National Natural Science Foundation of China, 31922090. HKU Seed Funding for Strategic Interdisciplinary Research Scheme. Hong Kong Research Grant Council Collaborative Research Fund, C5062‐21GF. Young Talent Program from Guangdong Province, 77010‐42150005. The Start‐up Grant from Sun Yat‐sen University, 77010‐12255006. Fundamental Research Funds for the Central Universities from Sun Yat‐sen University, 77010‐13130003. Innovation and Technology Fund (funding support to State Key Laboratory of Agrobiotechnology).

## Conflicts of Interest

The authors declare no conflicts of interest.

## Supporting information


**Data S1:** gcb70798‐sup‐0001‐Supinfo.pdf.

## Data Availability

The continuous soil respiration database is available at https://github.com/bpbond/cosore/releases. The FLUXNET 2015 database is available at https://fluxnet.org/data/fluxnet2015‐dataset/. The Ameriflux database is available at https://ameriflux.lbl.gov/. The MODIS product MCD12C1 is available at https://ladsweb.modaps.eosdis.nasa.gov/missions‐and‐measurements/products/MCD12C1. The ELM simulation products of soil respiration and its components used in this study are available at https://doi.org/10.17605/OSF.IO/2NAKH.

## References

[gcb70798-bib-0001] Aubrey, D. P. , and R. O. Teskey . 2009. “Root‐Derived CO2 Efflux via Xylem Stream Rivals Soil CO_2_ Efflux.” New Phytologist 184: 35–40. 10.1111/j.1469-8137.2009.02971.x. 19674328

[gcb70798-bib-0002] Baldocchi, D. , J. Tang , and L. Xu . 2006. “How Switches and Lags in Biophysical Regulators Affect Spatial‐Temporal Variation of Soil Respiration in an Oak‐Grass Savanna.” Journal of Geophysical Research: Biogeosciences 111: G02008. 10.1029/2005JG000063.

[gcb70798-bib-0003] Ballantyne, A. , W. Smith , W. Anderegg , et al. 2017. “Accelerating Net Terrestrial Carbon Uptake During the Warming Hiatus due to Reduced Respiration.” Nature Climate Change 7: 148–152. 10.1038/nclimate3204.

[gcb70798-bib-0004] Bloemen, J. , M. A. McGuire , D. P. Aubrey , R. O. Teskey , and K. Steppe . 2013. “Transport of Root‐Respired CO2 via the Transpiration Stream Affects Aboveground Carbon Assimilation and CO_2_ Efflux in Trees.” New Phytologist 197, no. 2: 555–565. 10.1111/j.1469-8137.2012.04366.x. 23057485

[gcb70798-bib-0005] Bloemen, J. , R. O. Teskey , M. A. McGuire , D. P. Aubrey , and K. Steppe . 2016. “Root Xylem CO2 Flux: An Important but Unaccounted‐For Component of Root Respiration.” Trees 30: 343–352. 10.1007/s00468-015-1185-4.

[gcb70798-bib-0006] Bond‐Lamberty, B. , A. Ballantyne , E. Berryman , et al. 2024. “Twenty Years of Progress, Challenges, and Opportunities in Measuring and Understanding Soil Respiration.” Journal of Geophysical Research: Biogeosciences 129: e2023JG007637. 10.1029/2023jg007637.

[gcb70798-bib-0007] Bond‐Lamberty, B. , B. Bond‐Lamberty , D. S. Christianson , et al. 2020. “COSORE: A Community Database for Continuous Soil Respiration and Other Soil‐Atmosphere Greenhouse Gas Flux Data.” Global Change Biology 26: 7268–7283. 10.1111/gcb.15353. 33026137 PMC7756728

[gcb70798-bib-0008] Brændholt, A. , K. Steenberg Larsen , A. Ibrom , and K. Pilegaard . 2017. “Overestimation of Closed‐Chamber Soil CO_2_ Effluxes at Low Atmospheric Turbulence.” Biogeosciences 14: 1603–1616. 10.5194/bg-14-1603-2017.

[gcb70798-bib-0077] Buchmann, N. 2000. “Biotic and Abiotic Factors Controlling Soil Respiration Rates in *Picea abies* Stands.” Soil Biology and Biochemistry 32: 1625–1635. 10.1016/S0038-0717(00)00077-8.

[gcb70798-bib-0009] Burrows, S. M. , M. Maltrud , X. Yang , et al. 2020. “The DOE E3SM v1.1 Biogeochemistry Configuration: Description and Simulated Ecosystem‐Climate Responses to Historical Changes in Forcing.” Journal of Advances in Modeling Earth Systems 12: e2019MS001766. 10.1029/2019ms001766.

[gcb70798-bib-0010] Carey, J. C. , J. Tang , P. H. Templer , et al. 2016. “Temperature Response of Soil Respiration Largely Unaltered With Experimental Warming.” Proceedings of the National Academy of Sciences 113: 13797–13802. 10.1073/pnas.1605365113. PMC5137763 27849609

[gcb70798-bib-0011] Chen, S. , J. Zou , Z. Hu , H. Chen , and Y. Lu . 2014. “Global Annual Soil Respiration in Relation to Climate, Soil Properties and Vegetation Characteristics: Summary of Available Data.” Agricultural and Forest Meteorology 198: 335–346. 10.1016/j.agrformet.2014.08.020.

[gcb70798-bib-0012] Cheruy, F. , J. L. Dufresne , S. Aït Mesbah , J. Y. Grandpeix , and F. Wang . 2017. “Role of Soil Thermal Inertia in Surface Temperature and Soil Moisture‐Temperature Feedback.” Journal of Advances in Modeling Earth Systems 9: 2906–2919. 10.1002/2017MS001036.

[gcb70798-bib-0013] Chu, H. , H. Ni , J. Ma , and Y. Shen . 2023. “What Is the Pathway That Determines the Diurnal Lag Time Between Soil Respiration and Soil Temperature?” Geoderma 431: 116344. 10.1016/j.geoderma.2023.116344.

[gcb70798-bib-0014] Conant, R. T. , M. G. Ryan , G. I. Ågren , et al. 2011. “Temperature and Soil Organic Matter Decomposition Rates ‐ Synthesis of Current Knowledge and a Way Forward.” Global Change Biology 17: 3392–3404. 10.1111/j.1365-2486.2011.02496.x.

[gcb70798-bib-0015] Cueva, A. , S. H. Bullock , E. López‐Reyes , and R. Vargas . 2017. “Potential Bias of Daily Soil CO2 Efflux Estimates due to Sampling Time.” Scientific Reports 7: 11925. 10.1038/s41598-017-11849-y. 28931840 PMC5607316

[gcb70798-bib-0016] Danger, M. , T. Daufresne , F. Lucas , S. Pissard , and G. Lacroix . 2008. “Does Liebig's Law of the Minimum Scale Up From Species to Communities?” Oikos 117: 1741–1751. 10.1111/j.1600-0706.2008.16793.x.

[gcb70798-bib-0017] Davidson, E. A. , and I. A. Janssens . 2006. “Temperature Sensitivity of Soil Carbon Decomposition and Feedbacks to Climate Change.” Nature 440: 165–173. 10.1038/nature04514. 16525463

[gcb70798-bib-0018] del Gutiérrez Arroyo, O. , and T. E. Wood . 2020. “Significant Diel Variation of Soil Respiration Suggests Aboveground and Belowground Controls in a Tropical Moist Forest in Puerto Rico.” Journal of Geophysical Research: Biogeosciences 125: e2019JG005353. 10.1029/2019jg005353.

[gcb70798-bib-0019] Doan, Q. V. , F. Chen , Y. Asano , et al. 2022. “Causes for Asymmetric Warming of Sub‐Diurnal Temperature Responding to Global Warming.” Geophysical Research Letters 49: e2022GL100029. 10.1029/2022gl100029.

[gcb70798-bib-0020] D'Odorico, P. , Y. He , S. Collins , S. F. De Wekker , V. Engel , and J. D. Fuentes . 2013. “Vegetation–Microclimate Feedbacks in Woodland–Grassland Ecotones.” Global Ecology and Biogeography 22: 364–379. 10.1111/geb.12000.

[gcb70798-bib-0021] Fekadu, G. , E. Adgo , D. T. Meshesha , et al. 2022. “Seasonal and Diurnal Soil Respiration Dynamics Under Different Land Management Practices in the Sub‐Tropical Highland Agroecology of Ethiopia.” Environmental Monitoring and Assessment 195: 65. 10.1007/s10661-022-10705-5. 36329265

[gcb70798-bib-0022] Ford, C. R. , J. McGee , F. Scandellari , E. A. Hobbie , and R. J. Mitchell . 2012. “Long‐ and Short‐Term Precipitation Effects on Soil CO2 Efflux and Total Belowground Carbon Allocation.” Agricultural and Forest Meteorology 156: 54–64. 10.1016/j.agrformet.2011.12.008.

[gcb70798-bib-0023] García‐Palacios, P. , T. W. Crowther , M. Dacal , et al. 2021. “Evidence for Large Microbial‐Mediated Losses of Soil Carbon Under Anthropogenic Warming.” Nature Reviews Earth & Environment 2: 507–517. 10.1038/s43017-021-00178-4.

[gcb70798-bib-0024] Görres, C.‐M. , C. Kammann , and R. Ceulemans . 2016. “Automation of Soil Flux Chamber Measurements: Potentials and Pitfalls.” Biogeosciences 13: 1949–1966. 10.5194/bg-13-1949-2016.

[gcb70798-bib-0025] Guenet, B. , J. Orliac , L. Cécillon , et al. 2024. “Spatial Biases Reduce the Ability of Earth System Models to Simulate Soil Heterotrophic Respiration Fluxes.” Biogeosciences 21: 657–669. 10.5194/bg-21-657-2024.

[gcb70798-bib-0026] Haaf, D. , J. Six , and S. Doetterl . 2021. “Global Patterns of Geo‐Ecological Controls on the Response of Soil Respiration to Warming.” Nature Climate Change 11: 623–627. 10.1038/s41558-021-01068-9.

[gcb70798-bib-0027] Han, G. , Y. Luo , D. Li , J. Xia , Q. Xing , and J. Yu . 2014. “Ecosystem Photosynthesis Regulates Soil Respiration on a Diurnal Scale With a Short‐Term Time Lag in a Coastal Wetland.” Soil Biology and Biochemistry 68: 85–94. 10.1016/j.soilbio.2013.09.024.

[gcb70798-bib-0028] Han, Y. , G. Wang , S. Zhou , W. Li , and L. Xiong . 2023. “Day–Night Discrepancy in Soil Respiration Varies With Seasons in a Temperate Forest.” Functional Ecology 37: 2002–2013. 10.1111/1365-2435.14358.

[gcb70798-bib-0029] Hanson, P. J. , N. T. Edwards , C. T. Garten , and J. A. Andrews . 2000. “Separating Root and Soil Microbial Contributions to Soil Respiration: A Review of Methods and Observations.” Biogeochemistry 48: 115–146. 10.1023/a:1006244819642.

[gcb70798-bib-0030] Hashimoto, S. , A. Ito , and K. Nishina . 2023. “Divergent Data‐Driven Estimates of Global Soil Respiration.” Communications Earth & Environment 4: 460. 10.1038/s43247-023-01136-2.

[gcb70798-bib-0031] Heinemeyer, A. , M. Wilkinson , R. Vargas , et al. 2012. “Exploring the “Overflow Tap” Theory: Linking Forest Soil CO_2_ Fluxes and Individual Mycorrhizosphere Components to Photosynthesis.” Biogeosciences 9: 79–95. 10.5194/bg-9-79-2012.

[gcb70798-bib-0032] Hirano, T. 2005. “Seasonal and Diurnal Variations in Topsoil and Subsoil Respiration Under Snowpack in a Temperate Deciduous Forest.” Global Biogeochemical Cycles 19: GB2011. 10.1029/2004gb002259.

[gcb70798-bib-0033] Hu, Z. , S. Liu , X. Liu , et al. 2016. “Soil Respiration and Its Environmental Response Varies by Day/Night and by Growing/Dormant Season in a Subalpine Forest.” Scientific Reports 6: 37864. 10.1038/srep37864. 27897252 PMC5126676

[gcb70798-bib-0034] Hursh, A. , A. Ballantyne , L. Cooper , M. Maneta , J. Kimball , and J. Watts . 2017. “The Sensitivity of Soil Respiration to Soil Temperature, Moisture, and Carbon Supply at the Global Scale.” Global Change Biology 23: 2090–2103. 10.1111/gcb.13489. 27594213

[gcb70798-bib-0035] Ito, A. , T. Hajima , D. M. Lawrence , et al. 2020. “Soil Carbon Sequestration Simulated in CMIP6‐LUMIP Models: Implications for Climatic Mitigation.” Environmental Research Letters 15: 124061. 10.1088/1748-9326/abc912.

[gcb70798-bib-0036] Jian, J. , M. K. Steele , R. Q. Thomas , S. D. Day , and S. C. Hodges . 2018. “Constraining Estimates of Global Soil Respiration by Quantifying Sources of Variability.” Global Change Biology 24: 4143–4159. 10.1111/gcb.14301. 29749095

[gcb70798-bib-0037] Kattge, J. , G. Bönisch , S. Díaz , et al. 2020. “TRY Plant Trait Database – Enhanced Coverage and Open Access.” Global Change Biology 26: 119–188. 10.1111/gcb.14904. 31891233

[gcb70798-bib-0038] Koven, C. D. , W. J. Riley , Z. M. Subin , et al. 2013. “The Effect of Vertically Resolved Soil Biogeochemistry and Alternate Soil C and N Models on C Dynamics of CLM4.” Biogeosciences 10: 7109–7131. 10.5194/bg-10-7109-2013.

[gcb70798-bib-0039] Kuzyakov, Y. , and W. Cheng . 2001. “Photosynthesis Controls of Rhizosphere Respiration and Organic Matter Decomposition.” Soil Biology and Biochemistry 33: 1915–1925. 10.1016/s0038-0717(01)00117-1.

[gcb70798-bib-0040] Kuzyakov, Y. , and O. Gavrichkova . 2010. “Review: Time Lag Between Photosynthesis and Carbon Dioxide Efflux From Soil: A Review of Mechanisms and Controls.” Global Change Biology 16: 3386–3406. 10.1111/j.1365-2486.2010.02179.x.

[gcb70798-bib-0041] Lasslop, G. , M. Reichstein , D. Papale , et al. 2010. “Separation of Net Ecosystem Exchange Into Assimilation and Respiration Using a Light Response Curve Approach: Critical Issues and Global Evaluation.” Global Change Biology 16: 187–208. 10.1111/j.1365-2486.2009.02041.x.

[gcb70798-bib-0042] Lawrence, D. M. , R. A. Fisher , C. D. Koven , et al. 2019. “The Community Land Model Version 5: Description of New Features, Benchmarking, and Impact of Forcing Uncertainty.” Journal of Advances in Modeling Earth Systems 11: 4245–4287. 10.1029/2018MS001583.

[gcb70798-bib-0043] Legendre, P. 2018. Model II Regression User's Guide, R Edition, (A Tutorial Within R Session, R Package Lmodel2). 10.32614/cran.package.lmodel2.

[gcb70798-bib-0044] Lehmann, J. , and M. Kleber . 2015. “The Contentious Nature of Soil Organic Matter.” Nature 528: 60–68. 10.1038/nature16069. 26595271

[gcb70798-bib-0045] Li, J. , J. Pei , E. Pendall , C. Fang , and M. Nie . 2020. “Spatial Heterogeneity of Temperature Sensitivity of Soil Respiration: A Global Analysis of Field Observations.” Soil Biology and Biochemistry 141: 107675. 10.1016/j.soilbio.2019.107675.

[gcb70798-bib-0046] Liang, G. , A. Stefanski , W. C. Eddy , et al. 2024. “Soil Respiration Response to Decade‐Long Warming Modulated by Soil Moisture in a Boreal Forest.” Nature Geoscience 17: 905–911. 10.1038/s41561-024-01512-3.

[gcb70798-bib-0047] Liu, Q. , N. T. Edwards , W. M. Post , L. Gu , J. Ledford , and S. Lenhart . 2006. “Temperature‐Independent Diel Variation in Soil Respiration Observed From a Temperate Deciduous Forest.” Global Change Biology 12: 2136–2145. 10.1111/j.1365-2486.2006.01245.x.

[gcb70798-bib-0048] Liu, Y. , R. Chen , C. Han , Z. Liu , Z. Yang , and Y. Zhao . 2024. “Diurnal Pattern and Characteristic of Soil Respiration and Net Ecosystem Carbon Exchange in Alpine Meadow Ecosystem on the Northeastern Qinghai‐Tibet Plateau.” Ecological Indicators 165: 112180. 10.1016/j.ecolind.2024.112180.

[gcb70798-bib-0049] Lloyd, J. , and J. A. Taylor . 1994. “On the Temperature Dependence of Soil Respiration.” Functional Ecology 8: 315. 10.2307/2389824.

[gcb70798-bib-0050] Luo, Y. , A. Ahlström , S. D. Allison , et al. 2016. “Toward More Realistic Projections of Soil Carbon Dynamics by Earth System Models.” Global Biogeochemical Cycles 30: 40–56. 10.1002/2015gb005239.

[gcb70798-bib-0051] Mahooti, M. 2025. “Sunrise Sunset, MATLAB Central File Exchange.” https://www.mathworks.com/matlabcentral/fileexchange/55312‐sunrise‐sunset.

[gcb70798-bib-0052] Makita, N. , Y. Kosugi , A. Sakabe , A. Kanazawa , S. Ohkubo , and M. Tani . 2018. “Seasonal and Diurnal Patterns of Soil Respiration in an Evergreen Coniferous Forest: Evidence From Six Years of Observation With Automatic Chambers.” PLoS One 13: e0192622. 10.1371/journal.pone.0192622. 29432465 PMC5809067

[gcb70798-bib-0053] Mencuccini, M. , and T. Hölttä . 2010. “The Significance of Phloem Transport for the Speed With Which Canopy Photosynthesis and Belowground Respiration Are Linked.” New Phytologist 185: 189–203. 10.1111/j.1469-8137.2009.03050.x. 19825019

[gcb70798-bib-0054] Mo, W. , M.‐S. Lee , M. Uchida , et al. 2005. “Seasonal and Annual Variations in Soil Respiration in a Cool‐Temperate Deciduous Broad‐Leaved Forest in Japan.” Agricultural and Forest Meteorology 134: 81–94. 10.1016/j.agrformet.2005.08.015.

[gcb70798-bib-0055] Oleson, K. , D. Lawrence , G. Bonan , et al. 2013. “Technical Description of Version 4.5 of the Community Land Model (CLM), NCAR Technical Note: NCAR/TN‐503+ STR.” National Center for Atmospheric Research (NCAR).

[gcb70798-bib-0056] Pastorello, G. , C. Trotta , E. Canfora , et al. 2020. “The FLUXNET2015 Dataset and the ONEFlux Processing Pipeline for Eddy Covariance Data.” Scientific Data 7: 225. 10.1038/s41597-020-0534-3. 32647314 PMC7347557

[gcb70798-bib-0057] Peng, S. , S. Piao , P. Ciais , et al. 2013. “Asymmetric Effects of Daytime and Night‐Time Warming on Northern Hemisphere Vegetation.” Nature 501: 88–92. 10.1038/nature12434. 24005415

[gcb70798-bib-0058] Peng, S. , S. Piao , T. Wang , J. Sun , and Z. Shen . 2009. “Temperature Sensitivity of Soil Respiration in Different Ecosystems in China.” Soil Biology and Biochemistry 41: 1008–1014. 10.1016/j.soilbio.2008.10.023.

[gcb70798-bib-0076] Phillips, C. L. , N. Nickerson , D. Risk , et al. 2011. “Interpreting Diel Hysteresis Between Soil Respiration and Temperature.” Global Change Biology 17: 515–527. 10.1111/j.1365-2486.2010.02250.x.

[gcb70798-bib-0059] Pries, C. E. H. , C. Castanha , R. C. Porras , and M. S. Torn . 2017. “The Whole‐Soil Carbon Flux in Response to Warming.” Science 355: 1420–1423. 10.1126/science.aal1319. 28280251

[gcb70798-bib-0060] Riley, W. J. , Q. Zhu , and J. Y. Tang . 2018. “Weaker Land–Climate Feedbacks From Nutrient Uptake During Photosynthesis‐Inactive Periods.” Nature Climate Change 8: 1002–1006. 10.1038/s41558-018-0325-4.

[gcb70798-bib-0061] Ruehr, N. K. , A. Knohl , and N. Buchmann . 2010. “Environmental Variables Controlling Soil Respiration on Diurnal, Seasonal and Annual Time‐Scales in a Mixed Mountain Forest in Switzerland.” Biogeochemistry 98: 153–170. 10.1007/s10533-009-9383-z.

[gcb70798-bib-0062] Ruehr, S. , T. F. Keenan , C. Williams , et al. 2023. “Evidence and Attribution of the Enhanced Land Carbon Sink.” Nature Reviews Earth & Environment 4: 518–534. 10.1038/s43017-023-00456-3.

[gcb70798-bib-0063] Rytter, R. M. 2013. “The Effect of Limited Availability of N or Water on C Allocation to Fine Roots and Annual Fine Root Turnover in Alnus Incana and *Salix viminalis* .” Tree Physiology 33: 924–939. 10.1093/treephys/tpt060. 23963409

[gcb70798-bib-0064] Savage, K. E. , and E. A. Davidson . 2003. “A Comparison of Manual and Automated Systems for Soil CO_2_ Flux Measurements: Trade‐Offs Between Spatial and Temporal Resolution.” Journal of Experimental Botany 54: 891–899. 10.1093/jxb/erg121. 12598560

[gcb70798-bib-0065] Schimel, J. P. , L. E. Jackson , and M. K. Firestone . 1989. “Spatial and Temporal Effects on Plant‐Microbial Competition for Inorganic Nitrogen in a California Annual Grassland.” Soil Biology and Biochemistry 21: 1059–1066. 10.1016/0038-0717(89)90044-8.

[gcb70798-bib-0066] Schneider, J. , L. Kutzbach , S. Schulz , and M. Wilmldng . 2009. “Overestimation of CO_2_ Respiration Fluxes by the Closed Chamber Method in Low‐Turbulence Nighttime Conditions.” Journal of Geophysical Research, Biogeosciences 114: 1–10. 10.1029/2008JG000909.

[gcb70798-bib-0067] Tang, J. , D. D. Baldocchi , and L. Xu . 2005. “Tree Photosynthesis Modulates Soil Respiration on a Diurnal Time Scale.” Global Change Biology 11: 1298–1304. 10.1111/j.1365-2486.2005.00978.x.

[gcb70798-bib-0068] Tang, J. , and W. J. Riley . 2021. “On the Modeling Paradigm of Plant Root Nutrient Acquisition.” Plant and Soil 459: 441–451. 10.1007/s11104-020-04798-5.

[gcb70798-bib-0069] Tang, X. , S. Fan , W. Zhang , S. Gao , G. Chen , and L. Shi . 2019. “Global Variability in Belowground Autotrophic Respiration in Terrestrial Ecosystems.” Earth System Science Data 11: 1839–1852. 10.5194/essd-11-1839-2019.

[gcb70798-bib-0070] Vargas, R. , and M. F. Allen . 2008. “Environmental Controls and the Influence of Vegetation Type, Fine Roots and Rhizomorphs on Diel and Seasonal Variation in Soil Respiration.” New Phytologist 179: 460–471. 10.1111/j.1469-8137.2008.02481.x. 19086292

[gcb70798-bib-0071] Warner, D. L. , B. Bond‐Lamberty , J. Jian , E. Stell , and R. Vargas . 2019. “Spatial Predictions and Associated Uncertainty of Annual Soil Respiration at the Global Scale.” Global Biogeochemical Cycles 33: 1733–1745. 10.1029/2019gb006264.

[gcb70798-bib-0072] Xia, J. , Y. Han , Z. Zhang , Z. Zhang , and S. Wan . 2009. “Effects of Diurnal Warming on Soil Respiration Are Not Equal to the Summed Effects of Day and Night Warming in a Temperate Steppe.” Biogeosciences 6: 1361–1370. 10.5194/bg-6-1361-2009.

[gcb70798-bib-0073] Xu, Z. , S. Tang , L. Xiong , et al. 2015. “Temperature Sensitivity of Soil Respiration in China's Forest Ecosystems: Patterns and Controls.” Applied Soil Ecology 93: 105–110. 10.1016/j.apsoil.2015.04.008.

[gcb70798-bib-0074] Zhu, Q. , W. J. Riley , C. M. Iversen , and J. Kattge . 2020. “Assessing Impacts of Plant Stoichiometric Traits on Terrestrial Ecosystem Carbon Accumulation Using the E3SM Land Model.” Journal of Advances in Modeling Earth Systems 12: e2019MS001841. 10.1029/2019ms001841.

[gcb70798-bib-0075] Zhu, Q. , W. J. Riley , J. Tang , and N. J. Bouskill . 2024. “Plant Responses to Elevated CO2 Under Competing Hypotheses of Nitrogen and Phosphorus Limitations.” Ecological Applications 34: e2967. 10.1002/eap.2967. 38469663

