# Supplementary material for: Widespread Higher Soil Respiration Rates at Nighttime Than Daytime Across Global Forest Ecosystems
Source: Glob Chang Biol. 2026 Mar 13;32(3):e70798. doi: 10.1111/gcb.70798 (PMC12988347; doi:10.1111/gcb.70798)
Supplement: Supplementary file 1 — Data S1: gcb70798‐sup‐0001‐Supinfo.pdf. [file GCB-32-e70798-s001.pdf]

Supplementary Information

Widespread higher soil respiration rates at nighttime than daytime across global forest ecosystems

Heng Huang, Jinyun Tang, Ben Bond-Lamberty, Peter B. Reich, Thomas W. Crowther, Jinshi Jian, Kun Zhang, Lingli Liu, Jin Wu*

*Corresponding author (Email: jinwu@hku.hk)

Figure S1. The diel difference in soil respiration (ΔR_s = nighttime R_s – daytime R_s, μmol m^{-2} s^{-1}) across different study sites in spring (a), summer (b), autumn (c), and winter (d), respectively. Positive values indicate higher nighttime values than daytime values. Dark-green and blue points indicate significant positive and negative ΔR_s at each site (P < 0.05), while brown points indicate statistically nonsignificant diel difference ($P \geq 0.05$). Error bars represent the stand error of the mean.

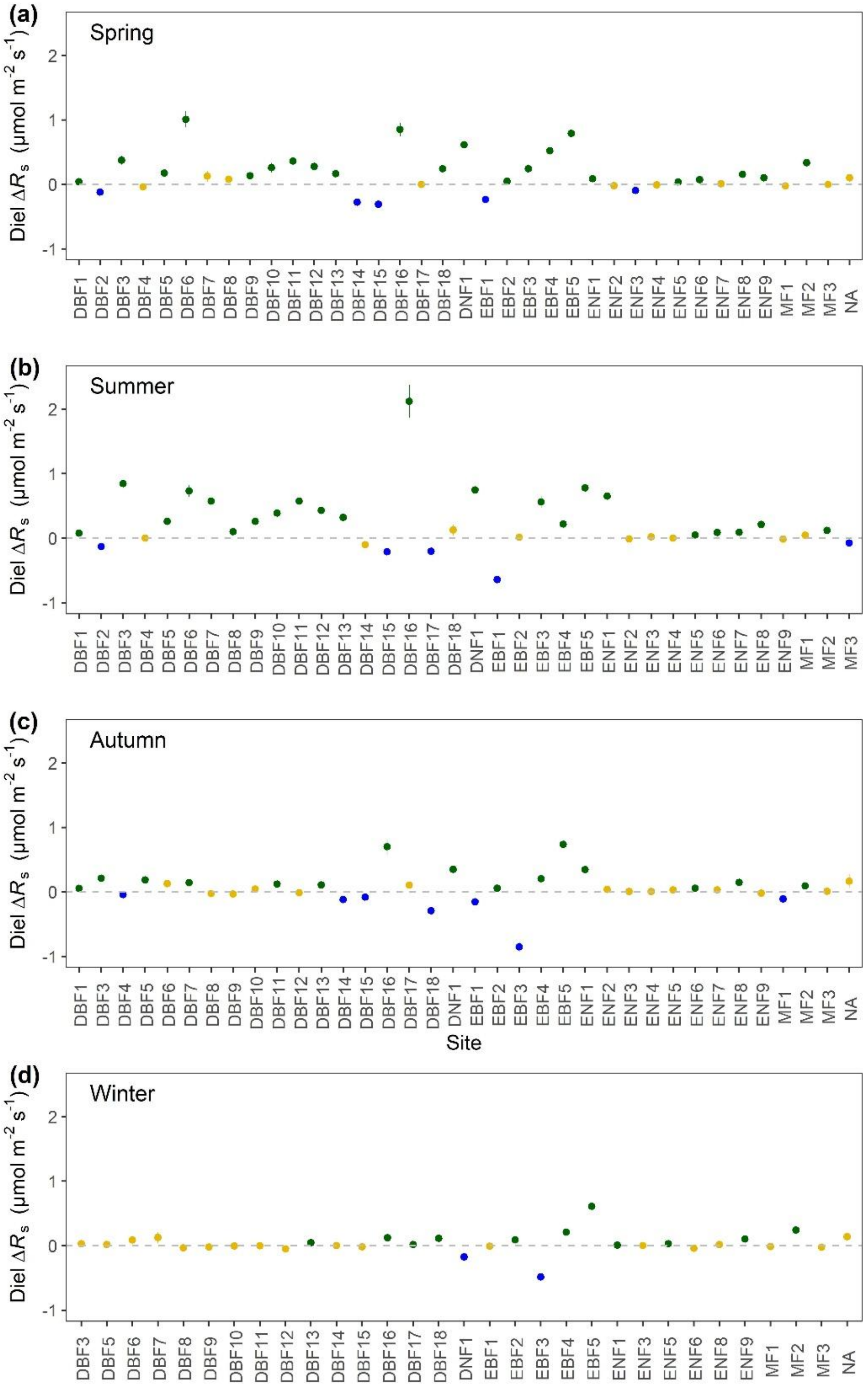

Figure S2. The diel difference in soil temperature (ΔT_s = nighttime T_s – daytime T_s, °C, a) and soil water content (ΔSWC = nighttime SWC – daytime SWC, m^3 m^{-3}, b) across different study sites. Positive values indicate higher nighttime values than daytime values. Dark-green and blue points indicate significant positive and negative diel differences at each site ($P < 0.05$), while brown points indicate statistically nonsignificant difference ($P \geq 0.05$). Error bars represent the stand error of the mean.

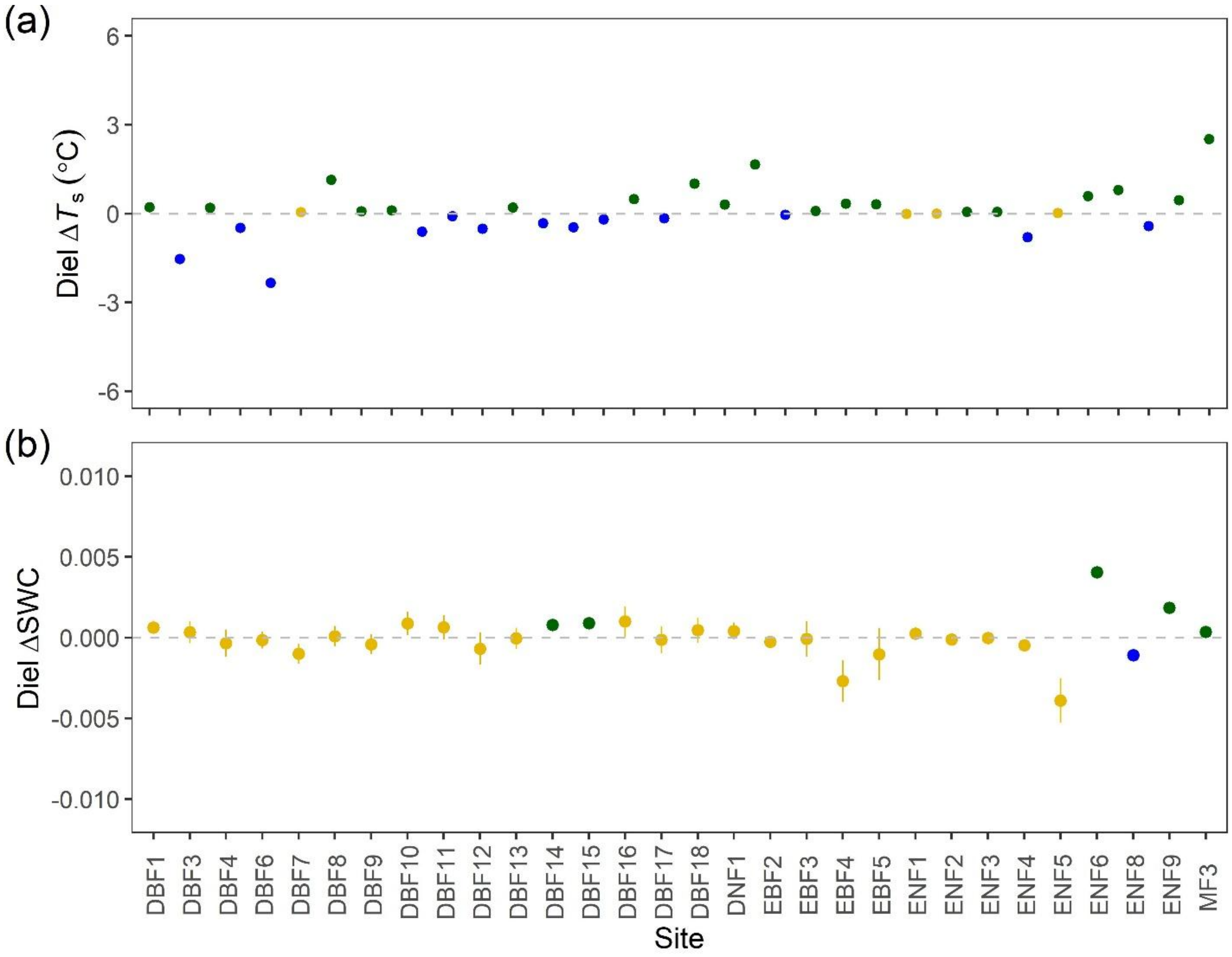

Figure S3. The relationship between soil respiration (R_s, μmol m^{-2} s^{-1}) and SWC (m^3 m^{-3}) across different study sites for daytime and nighttime periods, respectively. The LOWESS fitting curves and 95% confidence intervals are shown for both daytime and nighttime data.

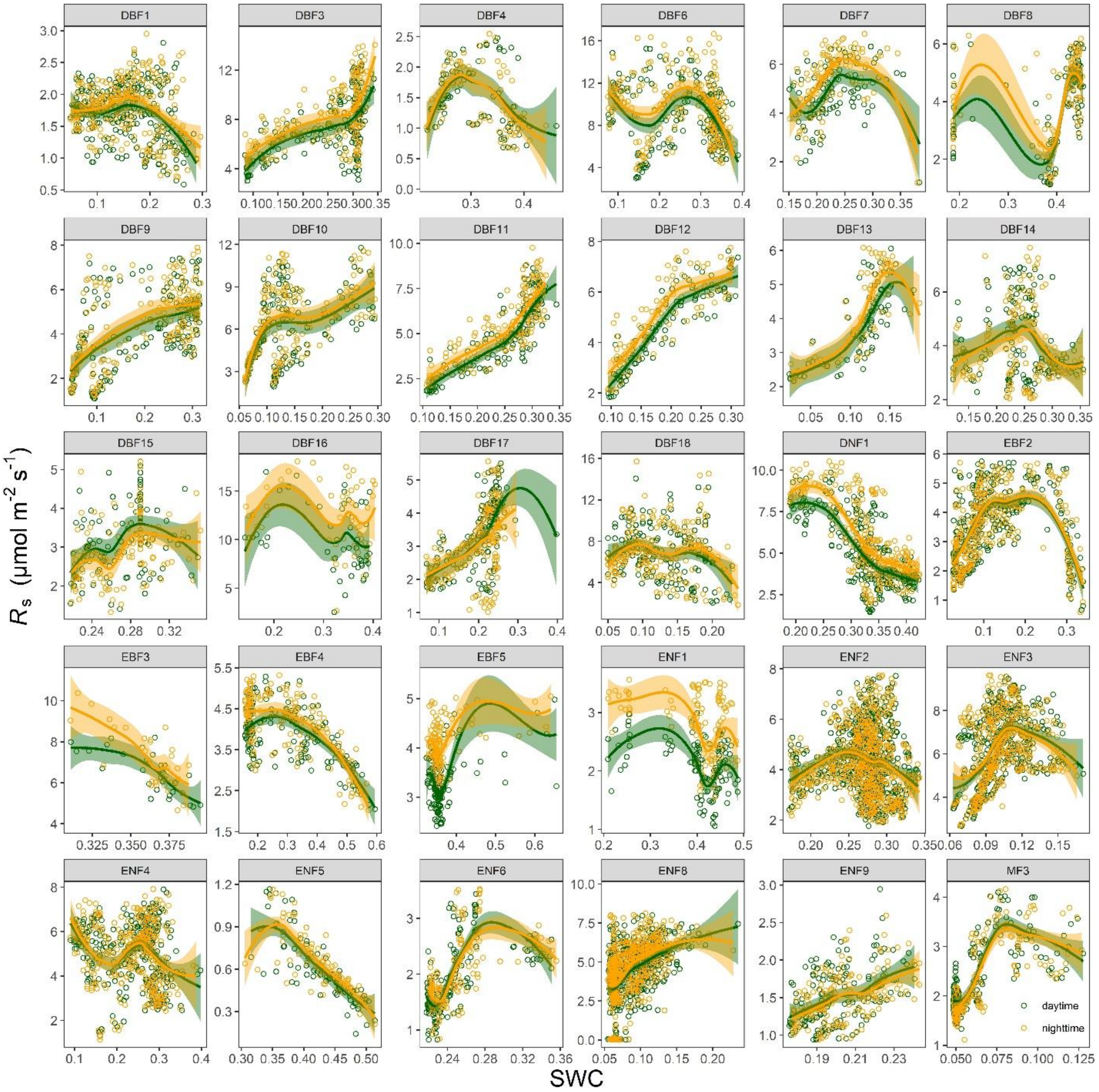

Figure S4. The diel difference in soil respiration adjusted to the site-specific mean temperature (ΔR_s^{adj} = nighttime R_s^{adj} – daytime R_s^{adj}, μmol m^{-2} s^{-1}) across different study sites. Positive values indicate higher nighttime values than daytime values. Dark-green and blue points indicate significant positive and negative diel differences at each site ($P < 0.05$), while brown points indicate statistically nonsignificant difference ($P \geq 0.05$). Error bars represent the stand error of the mean.

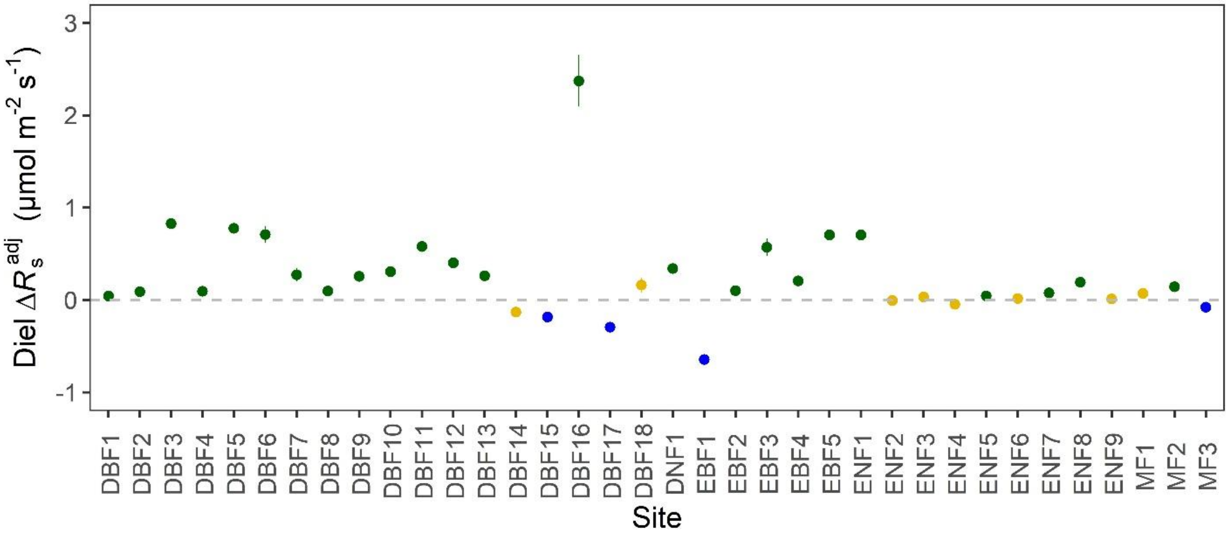

Figure S5. The daytime and nighttime relationships of Q_{10} with mean T_s (a) and mean SWC (b) across study sites, respectively.

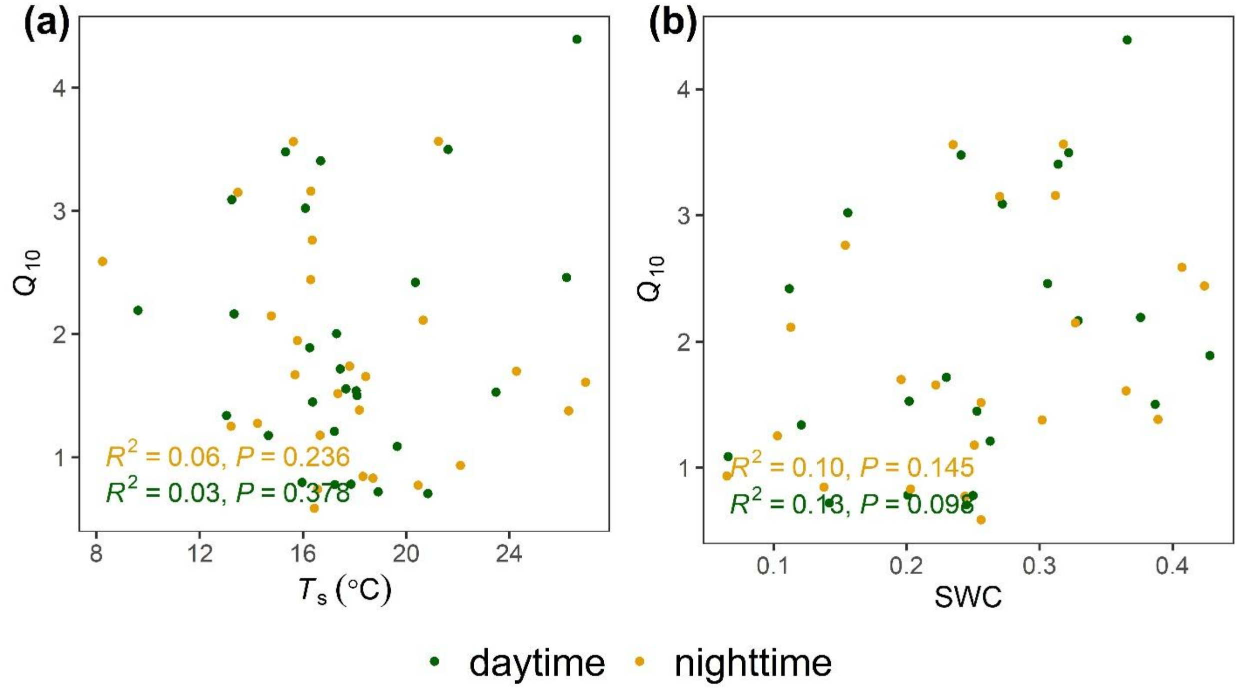

Figure S6. The comparison between daytime and nighttime root respiration (R_{root}, μmol m^{-2} s^{-1}) and soil heterotrophic respiration (R_h, μmol m^{-2} s^{-1}) from ELM. (a) The comparison between simulated daytime and nighttime R_{root} during 2005–2014 across study sites. **(b)** The comparison between simulated daytime and nighttime R_h during 2005–2014 across study sites. The dashed and solid lines represent the 1:1 relationships and linear regression results, respectively. Note that the RD configuration allows nighttime root growth through temporarily negative carbon storage pool, while the ECA configuration does not incorporate this process.

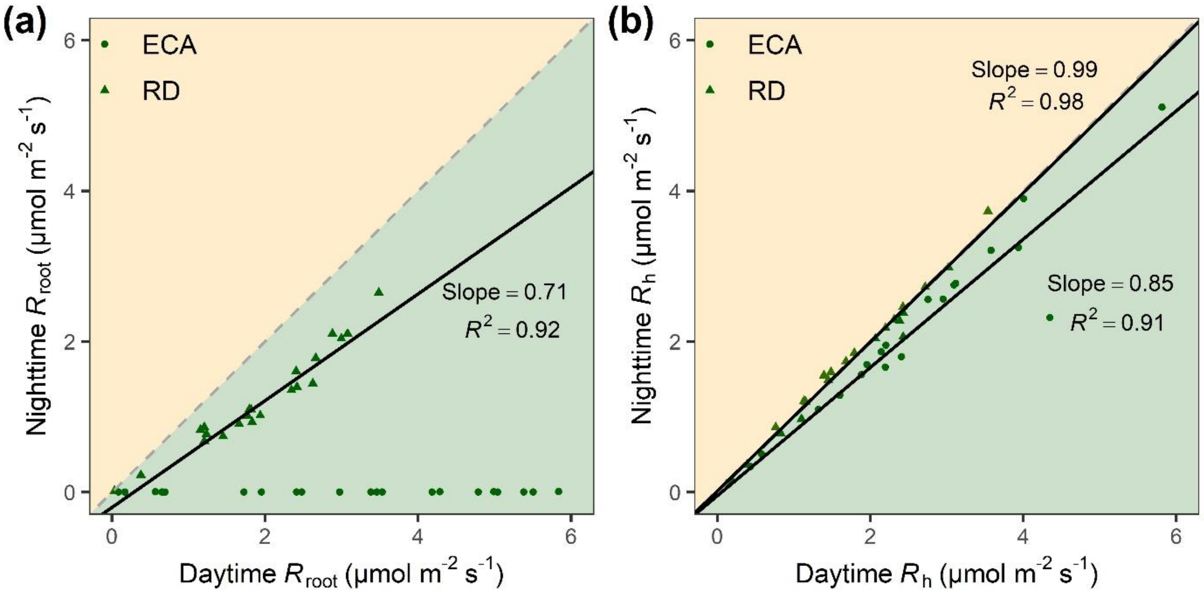

Figure S7. The relationship between soil respiration (R_s, μmol m^{-2} s^{-1}) and soil temperature (°C) for each study site during daytime and nighttime periods, respectively. Green and orange lines represent daytime and nighttime relationships, respectively.

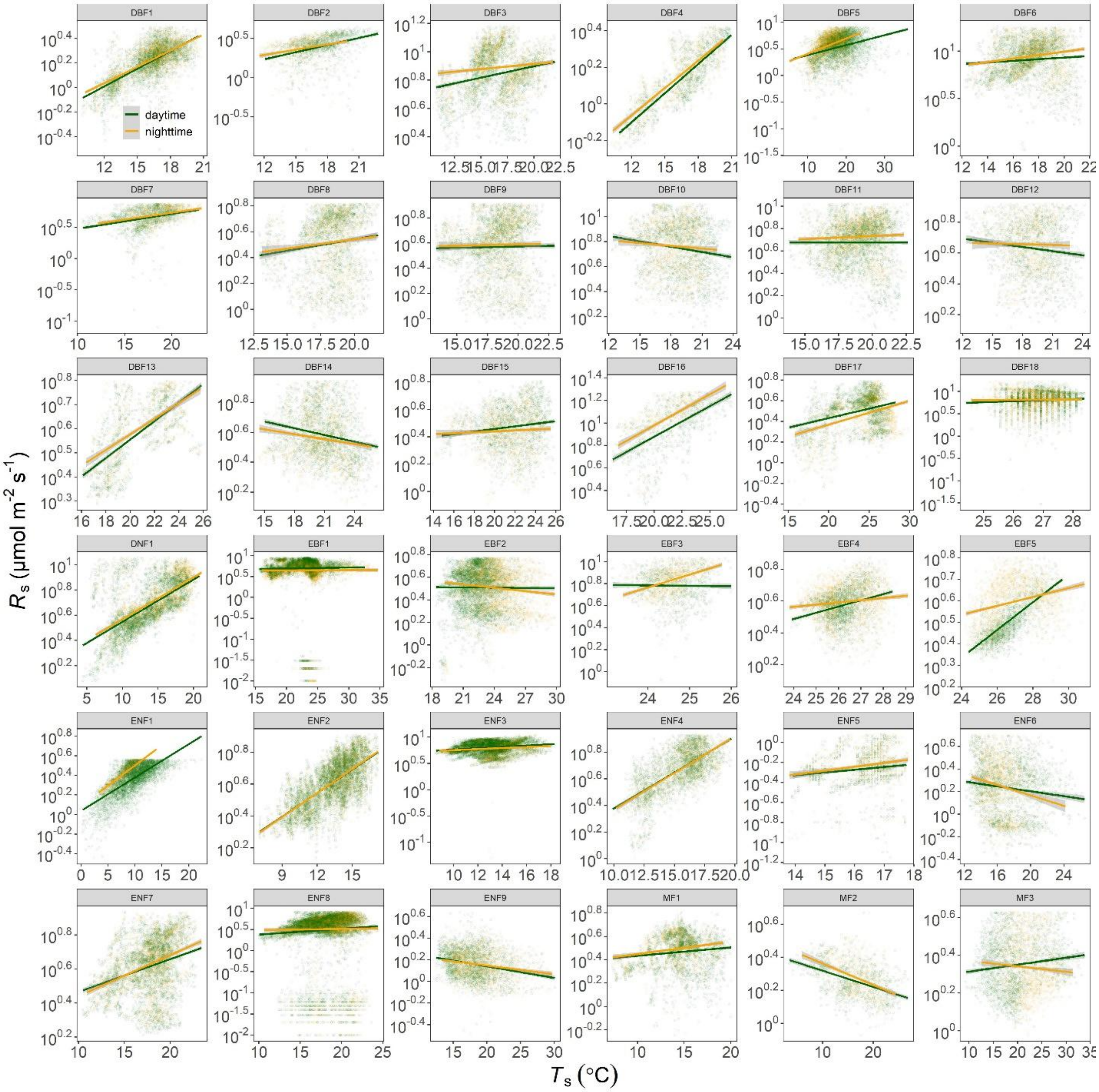

Figure S8. The bias in estimating nighttime $\boldsymbol{R_s}$ (µmol m^{-2} s^{-1}) using daytime $\boldsymbol{R_s}$-$\boldsymbol{T_s}$ relationships. Dark-green and blue points indicate significant positive and negative differences at each site ($P < 0.05$), while brown points indicate statistically nonsignificant difference ($P \geq 0.05$). Error bars represent the stand error of the mean.

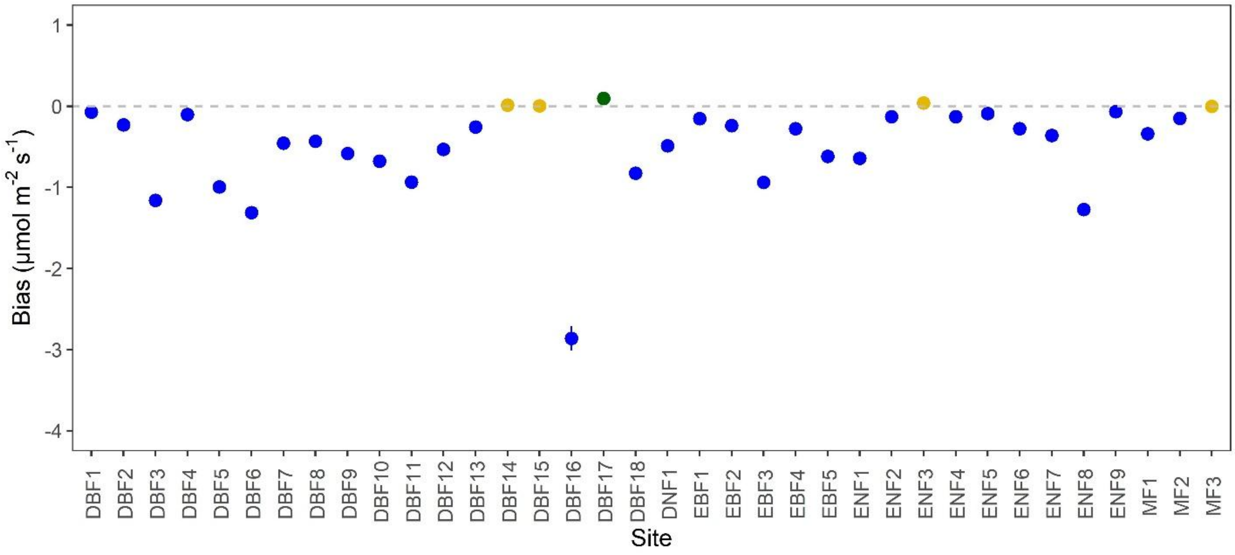

Figure S9. The relationships of daytime and nighttime R_s with daily GPP across different study sites. Solid and dashed lines represent nighttime and daytime relationships, respectively. The GPP data were obtained from the FLUXNET or AmeriFlux database.

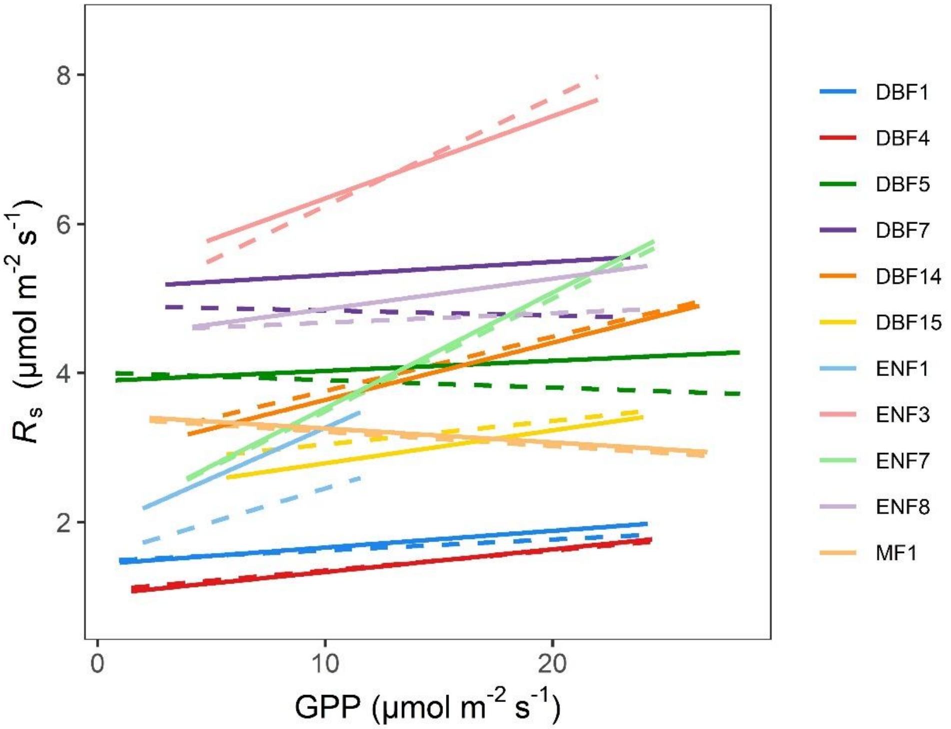

Figure S10. Changes in the Pearson correlation coefficient between daily *R*s and GPP as a function of a temporal lag between *R*s and GPP. The time lag specifies the number of days prior to the *R*s measurements that the GPP data were used for the correlation analysis.

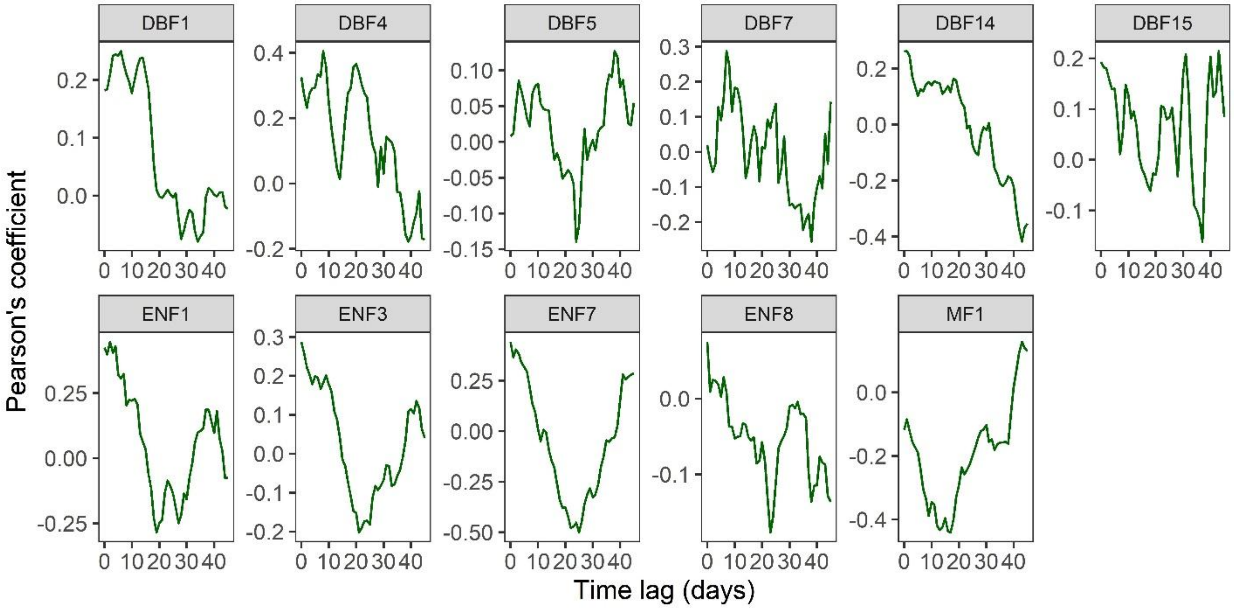

Figure S11. The comparison between daytime and nighttime root respiration (R_{root}, μmol m^{-2} s^{-1}) and soil heterotrophic respiration (R_h, μmol m^{-2} s^{-1}) from COSORE database. (a) The comparison between measured daytime and nighttime R_{root} across study sites. **(b)** The comparison between measured daytime and nighttime R_h across study sites. Each black point represents the site-specific mean value. The dashed and solid lines represent the 1:1 relationships and linear regression results, respectively.

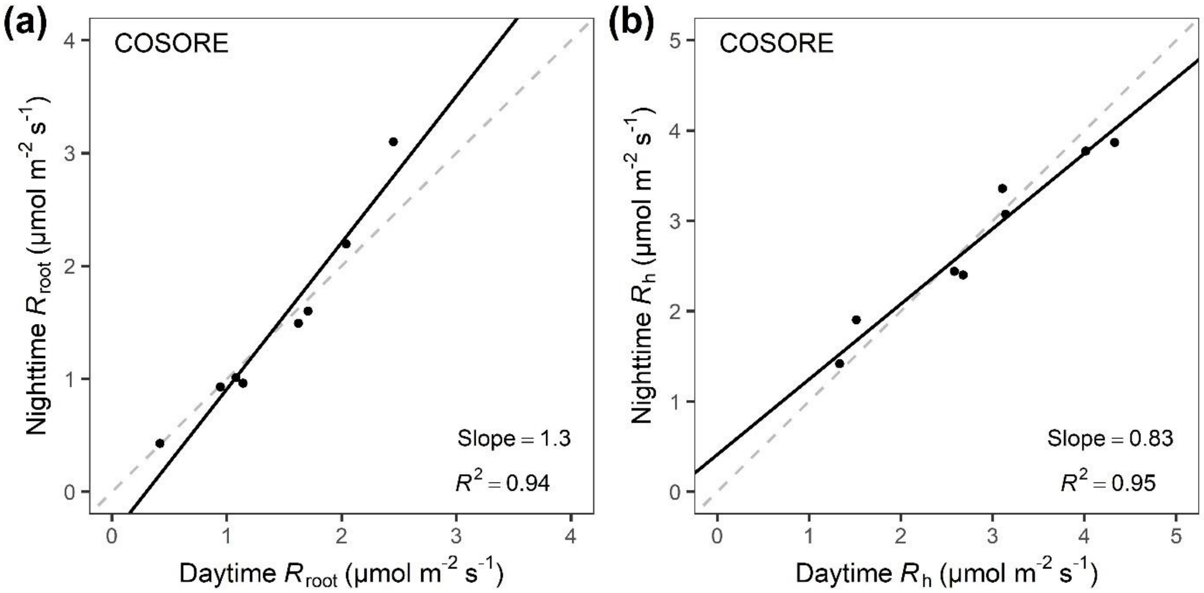

Table S1. Summary of diel difference (Δ = nighttime value – daytime value) in soil temperature (T_s, °C), soil water content (SWC), soil respiration (R_s, μmol m^{-2} s^{-1}), and parameters of R_s-T_s relationships across study sites.

Site ID	Dataset	Mean ΔT_s	Mean ΔSWC	Mean ΔR_s	Q_{10} (day, night)	$\log_{10}(R_{s0})$ (day, night)
DBF1	d20190504_SAVAGE_hf006-05	0.21	0.0006	0.08	3.02, 2.76	-0.57, -0.49
DBF2	d20190722_GORRES	-1.54	NA	-0.13	2, 1.67	-0.13, 0.02
DBF3	d20200212_KAYE_USW	0.19	0.0003	0.85	1.45, 1.18	0.58, 0.77
DBF4	d20190504_SAVAGE_hf006-03	-0.49	0.0002	0.00	3.4, 3.16	-0.74, -0.66
DBF5	d20190415_VARNER	-2.34	NA	0.26	1.54, 1.95	0.2, 0.12
DBF6	d20200212_KAYE_LSW	0.04	-0.0001	0.73	1.21, 1.52	0.76, 0.63
DBF7	d20190430_DESAI	1.14	-0.0010	0.57	1.72, 1.66	0.24, 0.29
DBF8	d20200212_KAYE_UNW	0.07	0.0001	0.10	1.5, 1.38	0.19, 0.26
DBF9	d20200212_KAYE_UNE	0.1	-0.0004	0.26	1.05, 1.05	0.53, 0.55
DBF10	d20200212_KAYE_LNE	-0.61	0.0009	0.39	0.72, 0.85	1.01, 0.9
DBF11	d20200212_KAYE_LNW	-0.1	0.0006	0.58	1, 1.14	0.68, 0.62
DBF12	d20200212_KAYE_USE	-0.51	-0.0007	0.43	0.81, 0.96	0.8, 0.69
DBF13	d20200212_ATAKA	0.2	-0.0000	0.32	2.42, 2.11	-0.21, -0.07
DBF14	d20190424_ZHANG_maple	-0.32	0.0008	-0.10	0.71, 0.78	0.9, 0.79
DBF15	d20190424_ZHANG_oak	-0.46	0.0016	-0.21	1.25, 1.08	0.27, 0.37
DBF16	d20190526_PENNINGTON	-0.2	0.0010	2.12	3.5, 3.56	-0.21, -0.12
DBF17	d20200328_UEYAMA_YAMASHIRO	0.49	-0.0001	-0.20	1.53, 1.7	0.06, -0.09
DBF18	d20190626_VARGAS	-0.16	0.0005	0.12	1.72, 1.14	0.17, 0.66
DNF1	d20200328_UEYAMA_TESHIO	1	0.0004	0.75	2.16, 2.15	0.21, 0.24
EBF1	d20190830_LIANG	0.31	NA	-0.64	1.06, 1.01	0.64, 0.64
EBF2	d20200228_RENCHON	1.65	-0.0003	0.01	0.98, 0.8	0.53, 0.74
EBF3	d20200207_GUTIERREZ_DEL_ARROYO	-0.04	-0.0001	0.56	0.92, 14.84	0.88, -2.05
EBF4	d20200109_HIRANO_PUF	0.09	-0.0027	0.22	2.46, 1.38	-0.45, 0.23
EBF5	d20200109_HIRANO_PDF	0.33	-0.0010	0.78	4.39, 1.61	-1.2, 0.04
ENF1	d20200122_BLACK	0.31	0.0002	0.65	2.19, 2.59	0.04, 0.09
ENF2	d20190610_SIHI_H1	-0.01	0.0002	-0.01	3.09, 3.15	-0.04, -0.05
ENF3	d20200108_JASSAL	-0.01	-0.0000	0.02	1.34, 1.25	0.64, 0.66
ENF4	d20190610_SIHI_H2	0.05	-0.0005	0.00	3.48, 3.56	-0.16, -0.18
ENF5	d20200120_CHANG	0.05	-0.0039	0.05	1.89, 2.44	-0.71, -0.86
ENF6	d20200114_CARBONE_SC_SAUCE	-0.8	0.0040	0.09	0.78, 0.59	0.42, 0.63
ENF7	d20200417_ARAIN_TP74	0.01	NA	0.09	1.56, 1.74	0.27, 0.2
ENF8	d20200417_ARAIN_TP39	0.59	-0.0011	0.21	1.37, 1.07	0.24, 0.46
ENF9	d20200114_CARBONE_SC_EMBUDO	0.79	0.0019	-0.01	0.78, 0.83	0.35, 0.31
MF1	d20190607_RUEHR	-0.43	NA	0.05	1.18, 1.28	0.36, 0.34
MF2	d20200114_CARBONE_JAMES	0.45	NA	0.12	0.8, 0.74	0.42, 0.49
MF3	d20200305_VARGAS	2.52	0.0003	-0.08	1.09, 0.94	0.28, 0.4
